# Sustainability in Health care by Allocating Resources Effectively (SHARE) 7: supporting staff in evidence-based decision-making, implementation and evaluation in a local healthcare setting

**DOI:** 10.1186/s12913-017-2388-8

**Published:** 2017-06-21

**Authors:** Claire Harris, Kelly Allen, Cara Waller, Tim Dyer, Vanessa Brooke, Marie Garrubba, Angela Melder, Catherine Voutier, Anthony Gust, Dina Farjou

**Affiliations:** 1 School of Public Health and Preventive Medicine, Monash University, Melbourne, VIC Australia; 2 Centre for Clinical Effectiveness, Monash Health, Melbourne, VIC Australia; 3 Clinical Information Management, Monash Health, Melbourne, VIC Australia

**Keywords:** Needs assessment, Needs analysis, Information needs, Evidence, Data, Capacity building, Decision-making, Implementation, Resource allocation, Disinvestment

## Abstract

**Background:**

This is the seventh in a series of papers reporting Sustainability in Health care by Allocating Resources Effectively (SHARE) in a local healthcare setting. The SHARE Program was a systematic, integrated, evidence-based program for resource allocation within a large Australian health service. It aimed to facilitate proactive use of evidence from research and local data; evidence-based decision-making for resource allocation including disinvestment; and development, implementation and evaluation of disinvestment projects. From the literature and responses of local stakeholders it was clear that provision of expertise and education, training and support of health service staff would be required to achieve these aims. Four support services were proposed. This paper is a detailed case report of the development, implementation and evaluation of a Data Service, Capacity Building Service and Project Support Service. An Evidence Service is reported separately.

**Methods:**

Literature reviews, surveys, interviews, consultation and workshops were used to capture and process the relevant information. Existing theoretical frameworks were adapted for evaluation and explication of processes and outcomes.

**Results:**

Surveys and interviews identified current practice in use of evidence in decision-making, implementation and evaluation; staff needs for evidence-based practice; nature, type and availability of local health service data; and preferred formats for education and training. The Capacity Building and Project Support Services were successful in achieving short term objectives; but long term outcomes were not evaluated due to reduced funding. The Data Service was not implemented at all. Factors influencing the processes and outcomes are discussed.

**Conclusion:**

Health service staff need access to education, training, expertise and support to enable evidence-based decision-making and to implement and evaluate the changes arising from those decisions. Three support services were proposed based on research evidence and local findings. Local factors, some unanticipated and some unavoidable, were the main barriers to successful implementation. All three proposed support services hold promise as facilitators of EBP in the local healthcare setting. The findings from this study will inform further exploration.

**Electronic supplementary material:**

The online version of this article (doi:10.1186/s12913-017-2388-8) contains supplementary material, which is available to authorized users.

## About SHARE

*This is the seventh in a series of papers reporting Sustainability in Health care by Allocating Resources Effectively (SHARE). The SHARE program is an investigation of concepts, opportunities, methods and implications for evidence-based investment and disinvestment in health technologies and clinical practices in a local healthcare setting. The papers in this series are targeted at clinicians, managers, policy makers, health service researchers and implementation scientists working in this context. This paper reports piloting of three of the four in-house staff support services to facilitate proactive use of evidence from local data; evidence-based decision-making for resource allocation including disinvestment; and development, implementation and evaluation of disinvestment projects.*

## Background

Monash Health, a large health service network in Melbourne Australia, sought to establish a program of disinvestment to improve patient outcomes by removing, reducing or restricting health technologies and clinical practices (TCPs) that were unsafe, ineffective or inefficient. The ‘Sustainability in Health care by Allocating Resources Effectively’ (SHARE) Program was established to investigate an organisation-wide, systematic, integrated, evidence-based approach to disinvestment in the context of resource allocation decisions.

The SHARE Program was undertaken by the Centre for Clinical Effectiveness (CCE), an in-house resource to facilitate Evidence Based Practice (EBP). An overview of the SHARE Program, a guide to the SHARE publications and further details about Monash Health (previously Southern Health) and CCE are provided in the first paper in this series [1] and a summary of the findings are in the final paper [2]. Funding was provided by the Victorian Department of Human Services (DHS) and Monash Health.

**Fig. 1:**
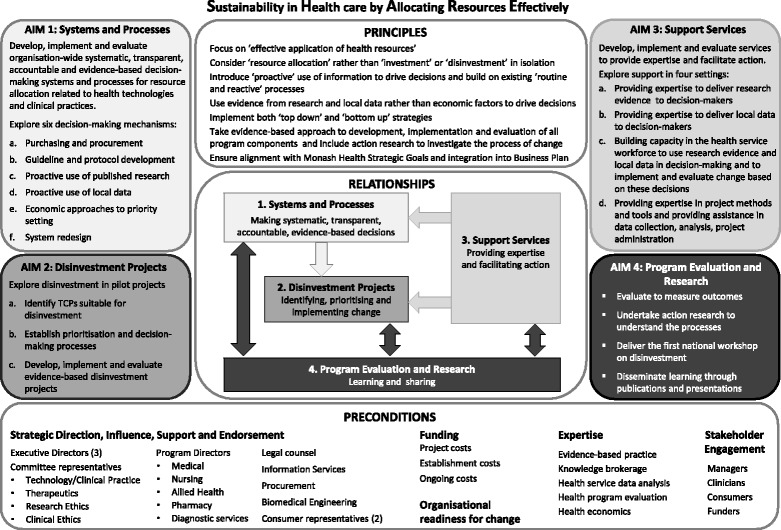
Overview of the SHARE Program Phase 2 (reproduced from Harris et at [6] with permission)

The SHARE Program was undertaken in two phases. Phase One explored concepts and practices related to disinvestment to understand the implications for a local health service and, based on this information, identified potential settings and methods for decision-making [3–5]. Phase Two developed, implemented and evaluated the proposed methods to determine which were effective, appropriate and sustainable at Monash Health [6]. The four aims of Phase Two are outlined in Fig. 1.

The first aim was to explore systems and processes for decision-making relating to TCPs. Objectives under this aim included investigation of methods for proactive access and utilisation of existing high quality research and health service data to initiate change [3]. The second aim was to pilot disinvestment projects [7].

Local research at Monash Health confirmed the findings of other studies that evidence from research and local data is not used systematically or proactively to drive decisions; that health service personnel usually lack the time, knowledge, skills and resources to access and identify the information they require and appraise it for quality and relevance; that clinicians charged with undertaking projects commonly do not know how to implement and evaluate change or manage projects effectively; and that projects are generally under-resourced [4, 6, 8–15]. It was clear that if the first two SHARE aims were to be achieved, services to support the proposed activities and build staff capacity and capability would be required [6].

**Fig. 2:**
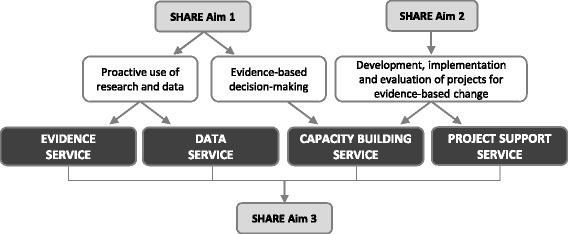
Relationship of support services to SHARE aims

The support services were intended to facilitate proactive use of evidence from research and local data; enable evidence-based decision-making (EBDM) for resource allocation including disinvestment; and aid development, implementation and evaluation of disinvestment projects. Four support services were proposed to meet these objectives: an Evidence Service, Data Service, Capacity Building Service and Project Support Service (Fig. 2). Piloting of these services became the third aim of the SHARE Program (Fig. 1).

There is a paucity of information about implementation of disinvestment recommendations, and a lack of understanding of the factors that influence the resource allocation process and the perspectives and experiences of healthcare staff undertaking disinvestment [16–20]. In-depth research at the health service level to address this gap and contribute to both the theory and practice of disinvestment has been proposed [18, 19, 21–24]. The fourth aim of the SHARE Program sought to achieve this (Fig. 1).

This paper reports on the Data Service, Capacity Building Service and Project Support Service; the Evidence Service is reported in Paper 8 of this series [25].

### Aims

The aim of this suite of projects was to develop, implement and evaluate the support services. The specific objectives of each service are outlined in Table 1.

**Table 1:** Objectives of the support services

Evidence service	
To provide research evidence to clinicians, managers and policy makers for use in decision-making. This will involve:	
▪ Disseminating high quality synthesised research evidence	
– Identifying sources of synthesised evidence	
– Establishing automated methods of capture	
– Collating evidence (eg effect, lack of effect, harm)	
– Categorising evidence by specialty and/or clinical setting	
– Prioritising based on user and health service needs	
– Translating into suitable formats based on user needs	
– Identifying relevant individuals or groups (generic or targeted)	
▪ Reviewing research literature to identify best practice for TCPs identified as potential opportunities for disinvestment from local data	
Data service	
To provide health service data to clinicians, managers and policy makers for use in decision-making. This will involve:	
▪ Investigating routinely-collected data to identify potential opportunities for disinvestment through	
– patterns of current practice (eg high volume; high cost; high rates of mortality, adverse events, readmission, reoperation, etc.; long length of stay)	
– variation in practice across Monash Health sites (eg high cost drug use at hospital A versus hospital B) or between Monash Health and other similar health services (caesarean section rates at Monash tertiary campus versus other tertiary campus)	
▪ Utilising routinely-collected local data to assess extent of use and relevance of TCPs identified as potential opportunities for disinvestment from reviews of research evidence	
Capacity Building Service	
To educate, train and support clinicians, managers and policy makers to use research and data in decision-making and then implement and evaluate these decisions in successful projects. This will involve provision of:	
▪ Education and upskilling programs in critical appraisal, data interpretation and organisational and clinical practice change (eg teaching modules, online resources, masterclasses)	
▪ Capacity building and support programs (eg clinical fellowships, mentoring programs)	
Project support service	
To provide methodological advice and practical project support to enable effective implementation and evaluation of evidence-based decisions. This will involve provision of:	
▪ Methodological advice and support	
▪ Assistance with project planning and administration	
▪ Assistance with data capture, entry and analysis	

The aim of this paper is to describe, explore and explain the process and outcomes of these pilot projects and the factors that influenced them.

### Research questions

What was current practice in accessing and using evidence for making, implementing and evaluating decisions at Monash Health?

What decisions were made and outcomes achieved in the piloting of the support services?

What factors influenced the decisions, processes and outcomes?

## Methods

### Design

#### Case study

The SHARE papers use a case study approach to address the limited understanding of resource allocation processes in health services, particularly regarding disinvestment [18, 19], and the lack of detailed reporting of implementation of change in the literature [26, 27]. Case studies allow in-depth, multi-faceted explorations of complex issues in their real-life settings [28] and facilitate development of theory and interventions [29]. The case study approach enables examination of the complex behaviours of, and relationships among, actors and agencies; and how those relationships influence change [30]. All these issues are intrinsic to the SHARE Program research questions.

All three case study approaches are used [31].

1. Descriptive: findings are reported in detail to describe events, processes and outcomes to enable replication when successful and avoidance or adaptation when unsuccessful
2. Exploratory: literature reviews, surveys, interviews, workshops and consultation with experts are used to explore what is known and identify actual, preferred and ideal practices
3. Explanatory: theoretical frameworks are used to understand and explain the events, processes and outcomes

#### Model for evidence-based change

**Fig. 3:**
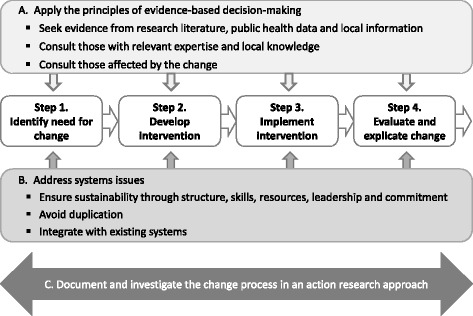
SEAchange model for Sustainable, Effective and Appropriate evidence-based change (adapted from Harris et al. [32] with permission)

Each support service was developed using the SEAchange model for Sustainable, Effective and Appropriate evidence-based change in health services (Fig. 3) [32]. The model involves four steps: identifying the need for change, developing an intervention to meet the need, implementing the intervention and evaluating the extent and impact of change. Each step is underpinned by the principles of evidence-based practice to ensure that the best available evidence from research and local data, the experience and expertise of health service staff and the values and perspectives of consumers are taken into account. Sustainability, avoidance of duplication and integration of new processes within existing systems are considered at each step.

Action research was undertaken based on the “*researcher as facilitator for change*” model defined by Meyer: researchers working explicitly with and for people rather than undertaking research on them [33, 34]. In this capacity, CCE staff were both the SHARE project team and the action researchers. An agenda item for ‘Learnings’ was scheduled at the beginning of every team meeting. Participants were invited to consider anything that had affected the project since the last meeting using the framework ‘what worked, what didn’t, why and how it could be improved’. Each issue, its effect on the project, and potential changes that would build on positive outcomes or remove or minimise future problems were discussed. The learnings and actions were documented; actions were assigned, given timeframes and followed up to ensure completion. Project team observations and reflections were used for ongoing improvements to the program components and implementation and evaluation processes.

*Identification of the need for change*

A literature review, surveys and interviews were undertaken to elicit information needs and barriers and enablers to EBDM, implementation and evaluation of change in local healthcare services [4, 25]. Data collection methods and sources and survey questions are listed in Additional file 1. Final interview and workshop notes were analysed thematically in Microsoft Word, Excel and Nvivo [35] by identification of emergent themes or categorisation according to the aims outlined in the individual project protocols.

*Development of the interventions*

Using the principles of evidence-based change, the SHARE team worked with stakeholders to synthesise the findings from published literature and local research, review and refine draft proposals, and develop frameworks and plans.

Strategic direction and governance decisions were made by the SHARE Steering Committee comprised of Executive Directors (Medical, Nursing, Support Services), Program Directors (Medical, Nursing, Allied Health, Pharmacy, Diagnostic Services), Committee chairs (Technology/Clinical Practice, Therapeutics, Human Research and Ethics, Clinical Ethics), Managers (Information Services, Clinical Information Services, Procurement, Biomedical Engineering, Research Services), Legal counsel and two Consumer representatives. Structured decision-making workshops were held at scheduled committee meetings. Discussion papers and background documents were provided beforehand, formal presentations introduced the workshops, and topics for discussion and decisions required were listed on the agenda. The deliberative process was informal within the structure of the agenda and decisions were based on consensus. Discussion, decisions and actions were documented in minutes. The project was endorsed by the Executive Management Team and Monash Health Board.

Modifications to the interventions were based on stakeholder feedback, evaluation findings and learnings from action research.

*Implementation*

Based on the SEAchange model of evidence-based change, planned implementation activities included engaging all stakeholders, identifying what is already known about practice change in the topic area from the literature and local knowledge, undertaking an analysis of local barriers and enablers, developing an implementation plan using strategies to minimise barriers and build on enablers, piloting and revising as required, and implementing in full [32]. Barriers and enablers to EBDM, implementation and evaluation at Monash Health were ascertained in the surveys and interviews noted above. Barriers and enablers to delivery of the pilot projects were determined from the evaluation and action research.

These were not all undertaken for each support service. Details are outlined in reports of the piloting processes below.

*Evaluation*

An evaluation framework and plan was developed for the overall SHARE Program and included evaluation domains, audience, scope, evaluation questions, outcomes hierarchy, sources of data, methods of collection and analysis, reporting and timelines [36]. Evaluation of the support services was addressed in the framework. A more detailed evaluation framework and plan was subsequently developed for the Capacity Building Service using the RE-AIM framework [37] and the UCSF-Fresno Medical Education Tool [38] which is discussed below and provided in Additional file 1.

### Explication of decisions, processes and outcomes

Factors that influenced decision-making for development of the support services were mapped to the relevant components of each intervention in a synthesis matrix adapted from Wallace et al. [8].

Factors that influenced processes and outcomes were identified using a theoretical framework for evaluation and explication of evidence-based innovations adapted for use in the SHARE Program [1]. Details of barriers and enablers, observable characteristics of the determinants of effectiveness, perceptions of participants and adopters, the process of change, findings from the action research process and other project team reflections were documented in minutes, reports, spreadsheets and templates for this purpose.

## Results and discussion

**Fig. 4:**
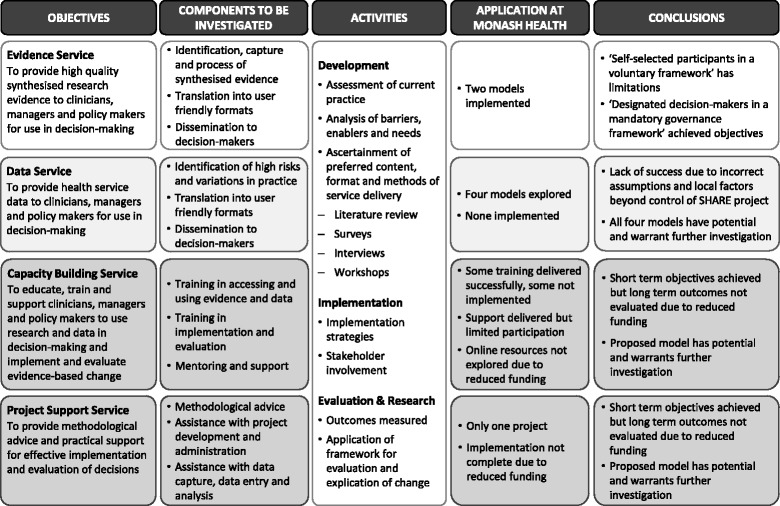
Overview of investigation of SHARE support services

An overview of the investigation of the SHARE support services is presented in Fig. 4.

The results of the literature search and the response rates of the surveys and interviews are reported in Additional file 1. Respondents included representatives of organisation-wide decision-making bodies, usually committee chairs; individuals with responsibility for resource allocation decisions as part of their role, mainly department or unit heads; members of project teams who had undertaken disinvestment activities; pharmacists and members of medication-focused committees; and staff members enrolling in EBP training courses or signing up to participate in the Evidence Service.

Data collected from these activities informed a range of research questions. Findings related to the research questions in this paper are provided in Additional file 1; findings related to topics not addressed here are reported in other SHARE publications [4, 7, 25]. Results from the literature review, 178 survey responses and 68 interviews were presented in detailed reports used for project decision-making and planning. They have been synthesised to address the research questions below.

Some of the planned activities were not completed due to reduction of funding in the final year of the SHARE Program resulting in shortening timelines; details and impact are discussed below.

Given the multiple components of this research, Results and Discussion are presented together to avoid repetition. Discussion of results in the context of the current literature follows the reporting of key findings.

### What was current practice in accessing and using evidence for making, implementing and evaluating decisions at monash health?

The survey and interview questions in this study focused on decision-making regarding resource allocation for TCPs and participants included all health professionals groups and health service managers. This is in contrast to the existing literature which focuses on use of evidence for clinical decisions and professional development in specified health professional populations [39–51]. Although the focus of the research questions and the participating decision-makers in these two contexts are slightly different, the findings are very similar.

The need for investigation of the differences in information-seeking behaviour between health professional groups has been identified [39]. This was not a primary objective of this study, however notable differences between medical, nursing, allied health and management/support groups emerged from the analysis. These are outlined in Additional file 1.

#### Sources and use of evidence

In the survey of staff who made decisions regarding allocation of resources (*n* = 118), 70% reported that they always or often included evidence from research in their decisions and all interviewees identified evidence from research as a key element of decision-making. However evidence was not defined in the survey or interview processes and the interviewee’s responses suggested that their understanding of evidence, evidence-based processes and critical appraisal was not consistent with current research definitions. We also know from previous work at Monash Health and elsewhere that although health service decision-makers report using evidence, they are often not aware of the different levels of evidence or how to assess quality [13, 52]. Therefore, although research evidence is reported as being used by most decision-makers, we cannot be sure that it was the best, most appropriate evidence for the decision.

Although the majority of survey respondents said they used research evidence always or often in their decisions, the most frequently used source of information for making decisions was colleagues (78%), followed by clinical practice guidelines (69%), original research (47%), systematic reviews (46%) and textbooks (35%) (Additional file 1). Similarly, committee representatives reported that they relied more on the knowledge and experience of *“experts”* on the committee who *“know the evidence”* than they did on finding research; and individual decision-makers generally drew on their existing knowledge more often than actively seeking the most up-to-date evidence noting that department heads *“know the research in their areas”*. Consulting with colleagues, contacts or experts as the main source of information is consistent with more recent reviews on this topic [12, 39, 44]. In contrast to most other studies where they were rated highly, textbooks were the least-used source of information at Monash Health [39, 40, 44, 47].

The most frequently used resource for finding information was the internet with 56% of respondents always or often using search engines such as Google, followed by electronic databases such as Medline (51%) and guideline websites (46%). This is also consistent with other studies [12, 39, 40, 42, 45, 48].

Interestingly, although 46% of respondents reported using systematic reviews for decision-making, only 27% always or often accessed The Cochrane Library.

In the survey of Monash Health staff enrolling to participate in the Evidence Service (*n* = 46), many respondents reported spending more than two hours for each of: finding (15/32), accessing (12/30) and appraising (12/30) research evidence for their decisions (Additional file 1).

Like evidence from research, local data was reported by interviewees (*n* = 68) to be an important consideration in decision-making, but there were no policies or processes to routinely ascertain or utilise data in committee or individual decisions. However local, state and national data were used in specific initiatives benchmarking local performance against appropriate counterparts in nursing and drug utilisation programs.

Only three of the staff involved in previous projects undertaking disinvestment (*n* = 10) accessed the literature for evidence regarding decisions to proceed with a project or development of the intervention, and only one accessed the literature for barriers and enablers to implementation. Routinely-collected local data was only used in two projects.

#### Knowledge, skills and confidence

Survey respondents (*n* = 118) were most confident finding evidence; 52% were very or quite confident in searching and 50% in accessing evidence (Additional file 1). There was slightly less confidence (46% very or quite confident) in using evidence in decisions and much less (34% very or quite confident) for appraising evidence. Whilst 34% of respondents reported that they were very or quite confident in appraising the evidence, this dropped considerably when aspects of quality appraisal were specified; for example assessment of study design (31%), influence of bias (28%), adequacy of sample size (24%), trustworthiness of an article (22%), and statistical tests and principles (14%). These results are also consistent with the findings of others [12, 15, 53]. Only 46% were very or quite confident in implementing evidence-based change (Additional file 1).

Decision-makers reported that the reasons they did not seek out research evidence were lack of time and the knowledge and skills to do so and those charged with undertaking projects reported that they lacked the appropriate knowledge and skills in implementation and evaluation.

### What decisions were made and outcomes achieved in the piloting of support services?

#### 1. Proactive use of evidence from local data (Data Service)

*1.1 Identification of the need for change*

Surveys and interviews with Monash Health staff found that use of data in decision-making was inconsistent and hindered by a number of barriers. The well-recognised generic factors such as lack of awareness; limited availability; poor quality; and lack of time, skills and resources to access, analyse and interpret data were present (Additional file 1). More specific local barriers included a perceived lack of consistent standards in the collection, production and dissemination of data leading to concerns about reliability and trustworthiness, and difficulties accessing data through the Monash Health intranet. Many of these issues were beyond the scope of the SHARE Program but some of those related to access and utilisation could potentially be addressed through a support service, referred to in this pilot as a ‘Data Service’.

Monash Health decision-makers often used local data to understand problems or develop solutions, but did not use it proactively to review current practice, seek opportunities for improvement or drive priority setting. This is consistent with the findings of others [52, 54], and the barriers, enablers, needs and proposal for proactive use of local data are also consistent with the current literature [11, 19, 52, 54–60].

Earlier SHARE work identified the potential to use targeted analysis of routinely-collected data to discover opportunities for disinvestment by identifying characteristics of TCPs where disinvestment might have the greatest impact such as high volume, high cost, extended length of stay, or high rates of adverse events; and investigating practice variation between campuses, departments or individuals to identify inappropriate or suboptimal practices suitable for disinvestment [3]. This might also be achieved through a Data Service.

*1.2 Development of an intervention*

Four models for a Data Service were proposed, however each faced insurmountable obstacles which are discussed below. When it became clear that a proposed model would be unworkable, a revised model based on the information available was investigated.

The initial proposal was for a service that would undertake three main activities.

- Interrogate routinely-collected data to identify potential disinvestment opportunities and communicate this information to appropriate decision-makers
- Respond to requests from decision-makers to assess local data related to potential disinvestment opportunities that had been identified from the research literature
- Provide training, advice and support in accessing and utilising local data to the Capacity Building and Project Support Services.

This proposal was based on assumptions that a Data Service could be delivered under similar conditions to the service delivered by CCE. The first assumption was that local data would be as readily accessible as published research. However at Monash Health there was no central repository of all the available data, data were held in a number of different sites across the organisation, linkages and coordination were limited, and no single person had access to all databases. The second assumption was that a single person could be engaged to undertake all the tasks proposed for the Data Service. Advice from internal and external experts in data utilisation was that staff working in this field, while usually highly skilled in one area, were unlikely to have the range of skills required. The need for this range of skills is echoed by others [60].

In consultation with the Monash Health Clinical Information Management (CIM) unit, the proposal was modified to consider only the data available within the CIM data warehouse and remove the training, support and knowledge brokerage activities. A position description was developed for a data analyst to be employed by CIM and seconded to the SHARE Program. No suitable candidate was identified from the recruitment process and the first proposal was withdrawn.

The second proposal was influenced by three changes within the organisation occurring at this time: the Executive Management Team was exploring a Knowledge Management Strategy; CCE had just established an Evaluation Service to provide expertise, support and training to health service staff; and a central resource to coordinate Monash Health projects was being considered. A knowledge brokerage model was proposed for the Data Service to complement and interface with these initiatives. A project officer with knowledge of all the data sources could liaise between decision-makers, data holders and data analysts. Methods and tools to facilitate these interactions would be developed.

A mapping exercise was conducted to identify the data available, methods of collection and storage, utilisation in decision-making, internal and external reporting, other forms of dissemination, strengths and weaknesses of the current system and opportunities for improvement. Thirty-eight databases were identified; only those most relevant to organisational decision-making for resource allocation for TCPs were explored. Interviews were conducted with representatives from ten departments that collected, maintained and shared data related to TCPs. The findings are reported in Additional file 1. As the data mapping exercise was concluding, Monash Health announced a project to extend the current CIM data warehouse to incorporate data from the other sources. The value of a brokerage model in this context was significantly reduced and the second proposal was also withdrawn.

The third proposal came from the funding body. When notified of the outcomes of the first two proposals, DHS requested that the SHARE team explore a Data Service that assisted committees to meet their data needs.

The CIM unit had a request form for committees to access data for decision-making, but it was used infrequently and often incorrectly. It was proposed that the SHARE team would design tools to help committees ‘ask the right question’ to facilitate their data requests. This concept was familiar to CCE staff who were skilled in helping clinicians and managers ‘ask the right question’ to ascertain research evidence from health publications, but would require training in the specifics of data requests. After development and piloting, the tools could be used more widely.

Members of the SHARE team consulted with representatives of the Monash Health committees previously identified as making decisions for resource allocation [4] to identify their ongoing and intermittent information needs. The committees indicated that they did not want assistance with access to or analysis of data. This model would not be effective under these circumstances and was withdrawn.

The fourth proposal built on recent developments in other areas. The Evidence Service had now also undergone several iterations and was screening high quality synthesised research to identify evidence with potential to change practice [25]. The CIM unit had also recently acquired a number of tools facilitating access to data; in particular the SQL (structured query language) database system that could report how many patients received a given intervention as indicated by an ICD-10 code. This meant that TCPs identified by the Evidence Service as potential disinvestment opportunities could be quantified in terms of numbers of cases, patient outcomes, cost, etc. The combination of research evidence and data could be used to identify, assess and prioritise potential disinvestment projects. A Data Service utilising the CIM tools to enhance the Evidence Service was proposed.

*1.3 Implementation*

The following steps were planned.

- Training of SHARE staff in accessing CIM data
- Developing resources to map Monash Health data processes
- Trialling the Data Service with examples from the catalogue of disinvestment opportunities developed in an earlier SHARE project [7]
- Adding Data Service functions into the Evidence Service processes for items tagged as disinvestment opportunities
- Linking Data Service functions into the Evidence Service reporting system
- Revising Evidence Service evaluation to include Data Service functions

Shortly after this work began, the funding was reduced and no further activities were undertaken due to the shortened timelines.

*1.4 Evaluation*

None of the proposed activities were implemented therefore no evaluation was undertaken.

#### 2. Evidence-based decision-making, implementation and evaluation (Capacity Building Service)

*2.1 Identification of the need for change*

Survey respondents and interviewees reported many barriers to searching for, accessing and appraising evidence; using it in decision-making; and implementing and evaluating change (Additional file 1). These were primarily lack of time, knowledge, skills, confidence and resources.

The need for education and training was highlighted by respondents. Self-paced online tutorials, workshops and short courses were the preferred methods (Additional file 1). Online resources were thought to be useful for *“time poor clinicians”* and courses were noted to have the benefits of *“group learning and discussion”* and *“peer support”* and that *“it can be easier to block out chunks of time”* to attend a workshop*.*

The need for ongoing support in addition to education and training was also acknowledged; for example *“follow up support to aid in using new skills”*. Some respondents felt “*isolated*” and noted the need for *“support from others who had done the same or similar work”*. Tailoring support to the needs of individuals or departments was emphasised.

The barriers, enablers and need for training and support are all consistent with the current literature [9–12, 15, 39, 40, 43, 45, 53, 58, 59, 61–69]. Several authors call for dedicated resources and in-house ‘resource centres’ to provide expertise; access to relevant methods and tools; and education, training and capacity-building [17, 59, 70–72]. The option proposed to address these issues was a ‘Capacity Building Service’.

*2.2 Development of an intervention*

The Pharmacy Department and four medication-related committees (Therapeutics, Medication Safety, Adverse Drug Reaction and High Cost Drugs) were chosen to pilot the Capacity Building Service due to the relevance of their roles to the SHARE aims and their interest in upskilling. All were involved in resource allocation decisions for purchase and/or use of pharmaceuticals and pharmaceutical-related equipment, the High Cost Drugs Working Party was undertaking disinvestment through a Therapeutics Equivalence Program [73], and Pharmacy management had requested training in EBP from CCE independent of the SHARE Program.

As the SHARE team had extensive experience with face-to-face teaching but no experience in delivering online content, the pilot program was offered as half-day interactive workshops. Five workshops were planned (Table 2).

**Table 2:** Activities of the Capacity Building Service and workshop learning objectives

Training workshops	
Interactive workshops to improve knowledge and skills	
▪ Evidence-based change process (½ day)	
– To understand the steps in developing, implementing and evaluating a change process	
– To apply the principles of evidence based practice to each step	
– To outline methods of collecting the information required to develop, implement and evaluate your project using this framework	
– To learn and share practical hints and tips for successful evidence-based change	
▪ Evidence-based practice (4 x ½ day)	
– To understand PICO elements and develop a searchable question	
– To learn the best research design to answer specific questions	
– To learn methods for searching health databases and undertake your own searches	
– To understand the role of chance, bias and confounding	
– To learn methods for critical appraisal and undertake appraisal exercises	
▪ Introduction to implementation (½ day)	
– To understand the principles of evidence-based implementation	
– To learn methods for identifying barriers and enablers and developing implementation strategies	
– To learn and share practical hints and tips for successful evidence-based implementation	
– To design an implementation plan for your project	
▪ Introduction to evaluation (½ day)	
– To understand evaluation: What? Why? When?	
– To understand evaluation frameworks and plans and data collection methods and sources	
− To consider the role of ethics in evaluation	
− To understand Program Logic Models	
▪ Using evidence in decision-making (1½ hours)	
– To consider the deliberation process and the role of decision-making criteria	
– To discuss the principles of evidence-based decision-making (EBDM)	
– To understand the implications of research design, level of evidence, quality, applicability, lack of evidence	
– To apply the learnings in worked examples	
– To be introduced to resources and services that support EBDM	
Problem solving/support sessions	
Rotating 4 weekly series of open workshops to provide ongoing support to workshop participants undertaking projects.	
▪ Week 1: Finding and appraising evidence and interpreting results	
▪ Week 2: Planning and implementing projects	
▪ Week 3: Evaluating programs and projects	
▪ Week 4: Developing guidelines and protocols	
Online resources/teaching (to be sourced or developed)	
▪ Electronic workbook	
▪ PowerPoint presentation/s	
▪ Self-assessment quizzes	

To provide ongoing support to workshop attendees, follow up sessions addressing evidence synthesis, project planning and implementation, evaluation and guideline development were offered on a rotating 4-weekly cycle (Table 2). Participants could seek feedback and assistance from CCE staff in the relevant topic area and share learnings and develop networks with colleagues.

It was proposed that existing online courses in EBP be identified, appraised and assessed for applicability at Monash Health and suitable resources be promoted via the CCE website.

*2.3 Implementation*

Pharmacy staff and members of the related committees received an email invitation to participate in any of the workshops. To promote the program, an introductory talk on EBP was held at a routine Pharmacy meeting; 37 staff members attended. In addition, staff involved in SHARE pilot disinvestment projects were invited to participate [7].

The first four workshops were delivered as planned. Twenty-two participants completed one or more of the courses: eleven from Pharmacy, four nurses, one allied health professional and six who did not specify their discipline. (Additional file 1). Half of the participants attended more than one workshop.

Existing CCE workshop materials were used but were customised to include pharmaceutical-related examples and exercises in the EBP sessions and allow participants to workshop their own projects in the Evidence-based change and Implementation sessions. After two of the EBP workshops, participants were sent a simple online revision quiz to consolidate their learning.

The fifth workshop on using evidence in decision-making was targeted at executives, program directors and committee members who made decisions based on information provided to them by others and did not search for or appraise evidence themselves. This workshop was not delivered due to the shortened SHARE timelines.

Only two participants attended the follow up sessions in the first 2 months. The program was discontinued and no evaluation was undertaken.

The concept of online versions of the material covered in the workshops was well received by participants. Many potentially suitable web-based resources were identified; however assessment of quality and applicability was not achieved within the shortened timelines.

*2.4 Evaluation*

Evaluation was undertaken using the RE-AIM framework of Reach, Effectiveness, Adoption, Implementation and Maintenance [37]. The findings are reported in detail in Additional file 1. The participant numbers for each activity were small, limiting the ability to draw conclusions; however general observations can be made.

Reach: The number of participants per workshop ranged from seven to eleven. The sessions were designed to accommodate up to 16 participants, so were well below capacity. These small numbers were probably also below the critical mass required to sustain the ongoing support sessions. Offering these courses to a wider audience may have resulted in greater utilisation of both the workshops and follow up support program.

Effectiveness: Evaluation immediately after workshops showed participants’ confidence improved in all aspects of the evidence-based change process and the concepts of EBP, implementation and evaluation. Self-perceived knowledge in aspects of implementation and evaluation also improved. Rather than relying on self-reported knowledge in EBP, the UCSF-Fresno Medical Education Tool [38] was adapted to multi-choice format and administered before and after the workshops. Only minor improvements in knowledge were recorded. This may be due to a ceiling effect, as participants’ baseline results (66%) were much higher than those in previous studies (17% to 54%) [74, 75], or that this version may not be as valid or reliable as other adaptations. Evaluation was also undertaken at 3 months post-workshop. Six of the seven participants of the Evidence-based change workshop responded; a further increase in confidence was noted in each category. Only five participants from each of the EBP (*n* = 11) and Implementation workshops (*n* = 8) responded; making it difficult to draw conclusions. Results for most outcomes measures were greater than baseline, but many were slightly less that immediately post-workshop. The 3 month survey was not administered for the Evaluation workshop due to the shortened timelines.

Adoption: Due to the reduced timelines the service was not expanded beyond the target audience.

Implementation: Four of the workshops and the follow up support sessions were delivered as planned. The fifth workshop and ascertainment and promotion of online resources were not undertaken due to the reduced timelines. Participants reported high rates of satisfaction and noted that the workshops met or exceeded their expectations. The online revision quizzes were not formally evaluated but were well-accessed and several participants provided positive feedback. General feedback and suggestions for improvement were invited; these are outlined in Additional file 1.

Maintenance: The program was discontinued due to the reduced timelines.

#### 3. Development, implementation and evaluation of disinvestment projects (Project Support Service)

*3.1 Identification of the need for change*

As noted above, Monash Health respondents were very clear about the barriers they faced, and their detailed responses also included specific suggestions to address them such as tailoring support to individual cases, enabling access to experts, providing practical assistance in computer skills and accessing and using data, and obtaining extra non-clinical time to implement and evaluate projects (Additional file 1).

The current literature also notes these specific needs for adequate and appropriate resources [13, 17, 76–78] including funding [76, 79–82]; time [56, 57, 61, 83–85]; administrative support [86]; and a range of appropriate expertise, methods and tools [18, 57–59, 61, 64, 70, 80, 87, 88]. A ‘Project Support Service’ to provide expertise and practical assistance to project staff in aspects of project management, planning, implementation and evaluation was proposed.

*3.2 Development of an intervention*

The pilot Project Support Service was developed to assist the clinical teams undertaking SHARE disinvestment pilot projects [7]. The nature and amount of guidance and support would be determined by the needs of individual projects. The service would be provided by CCE staff with the relevant expertise. As a range of skills were likely to be required for implementation and evaluation in different circumstances, the Project Support Service team would also liaise with other relevant experts such as Monash Health business managers and data custodians, university statisticians and the SHARE consultant health economist.

*3.3 Implementation*

Four applications were accepted as SHARE pilot disinvestment projects [7].

The first project had been approved by the Monash Health Technology/Clinical Practice Committee (TCPC) and approved and funded by the Victorian Policy and Advisory Committee on Technology (VPACT). It was withdrawn from the SHARE pilot process by the clinical project leaders before any significant assistance had been provided, however several discussions regarding potential support had been undertaken.

The clinicians leading the second project initially requested help to design their implementation and evaluation plans, however the project did not reach this stage. Although it was initiated in response to a recommendation in a new national guideline, the clinicians subsequently questioned the evidence underpinning this recommendation. CCE staff provided expertise and support in assessing the methods of guideline development, retrieving the evidence used to formulate the recommendation, searching for additional evidence, critically appraising identified studies and explaining and discussing study design and statistical analysis with the clinicians. The clinical project team also attended three Capacity Building Service workshops on evidence-based change, implementation and evaluation. Unrelated to considerations regarding the evidence, the clinicians finally decided that the practice put forward for disinvestment was not routinely performed at Monash Health and the project was withdrawn.

The third project had potential as a disinvestment activity but was not well defined. In order to establish the exact nature of the problem and design an appropriate intervention, the initial Project Support Service activities involved reviewing the literature and meeting with relevant staff to understand the local implications. An investigation of patterns of overuse and inappropriate practices was planned, which would have provided the additional benefit of an opportunity to pilot aspects of a Data Service. However this project was also withdrawn when it became clear that external factors would prevent it from being achieved within the original SHARE timelines (this decision was made prior to reduction of funding in the final year).

The fourth project had also been approved by the TCPC and VPACT. The clinical project team attended the Capacity Building Service workshops on evidence-based change, implementation and evaluation and worked with Project Support Service staff to develop an implementation plan, an evaluation and reporting framework, and a cost-comparison plan [7]. The Project Support Service provided direct assistance in identifying indicators to meet VPACT requirements; designing and developing a data collection tool and purpose-built Microsoft Access database; training in use of Microsoft Access, data entry and data analysis; and ongoing problem solving. Project Support Service staff also liaised on behalf of the clinical team with the TCPC and VPACT to streamline reporting, CIM to determine applicability of existing routinely-collected data and methods of access, Health Information Services to confirm that the new data collection tool would meet requirements for documents included in the medical record, a statistician to ensure appropriate methods were used, the Quality Unit regarding development of a protocol for referral and treatment of eligible patients, and the SHARE health economist for advice on the cost-comparison plan.

*3.4 Evaluation*

Due to the shortened timelines, many of the evaluation activities were not undertaken. The lack of formal evaluation, the variations in nature and intensity of support required by different projects, and the small number of SHARE pilot projects limit the ability to draw conclusions, however we can describe some key elements.

It was anticipated that a selection of activities related to project management, planning, implementation and evaluation would be provided by the Project Support Service, depending on the needs of individual projects (Table 3). We experienced the two extremes of level of assistance required and a third unpredicted outcome. The first project was withdrawn before any assistance had been provided, however the clinicians involved had expressed a need for assistance with implementation and evaluation. In contrast, the fourth project required support in all the anticipated areas. In the second and third projects, almost no support was required in the anticipated areas as the projects were not implemented. However the clinicians needed considerable assistance in unexpected areas such as reviewing evidence and data, determining the nature and scope of the problem, and clarifying the intervention; none of which had been anticipated for a service to support project delivery which would occur after decision-making. The clinicians in the first and fourth projects had accessed this assistance from CCE and CIM when developing their applications as required by the TCPC process [13].

**Table 3:** Activities of the Project Support Service

Stage of project	Activities	Proposed	SHARE projects			
			1	2	3	4
Decision-making and project development	Searching literature			✓	✓	
	Appraisal of evidence			✓	✓	
	Analysis of local data				✓	
	Determination of nature and scope of problem			✓	✓	
	Clarification of the intervention			✓	✓	
	Analysis of feasibility and risk				✓	
Project planning	Confirmation and documentation of scope, objectives, background, etc	✓	✓	✓	✓	✓
	Identification of needs of clinical project team	✓		✓	✓	✓
	Identification of stakeholders	✓				✓
Project management	Confirmation and documentation of governance processes	✓				✓
	Establishment of management and administration systems and processes	✓				✓
Implementation planning	Capture and analysis of barriers and enablers	✓				✓
	Identification of strategies to address barriers and enablers	✓				
	Development of implementation plan (including communication plan)	✓				✓
	Liaison with committees/departments for authorisation of practice change	✓				✓
	Liaison with committees/departments for authorisation of documentation	✓				✓
Evaluation planning	Development of evaluation framework and plan	✓				✓
	Development of costing/economic evaluation plan	✓				✓
	Identification of relevant tools	✓				✓
Development of data collection systems	Liaison with Health Information Management to determine codes	✓				✓
	Liaison with Clinical Information Management to access patient data	✓				✓
	Liaison with data analysts, statistician, health economist, other experts	✓				✓
	Development of data collection tools	✓				✓
	Development of electronic database (eg Access or Excel)	✓				✓
	Training project workers in use of database programs	✓				✓
Evaluation	Assistance with data entry	✓				✓
	Assistance with data cleaning	✓				
	Assistance with data analysis	✓				
Reporting	Development of reporting schedule	✓				✓
	Assistance with reporting	✓				

Each of the four clinical project teams acknowledged their lack of skills and experience in using evidence in decision-making, implementation and evaluation (Table 3), they were appreciative that support was available and were willing to seek help and accept guidance.

Although the fourth project was only partly implemented when the SHARE Program concluded prematurely, the clinicians agreed to complete an assessment of ‘what worked, what didn’t and how could things be improved?’ for their project overall. Expertise from CCE staff, practical support in development of the evaluation plan and design of a Microsoft Access database, and assistance with data entry and reporting were noted as positive factors (Additional file 1).

These outcomes highlight four points which are consistent with the authors’ previous experience in a wide range of health service projects, earlier SHARE work [4, 7] and the findings of others [8, 9, 12, 15, 76, 89, 90] and reinforce the need for Project Support Services within a local health service.

- Decisions to proceed with a project to implement change are often made without consideration of research evidence and local data and are not well-defined in terms of the intervention, practitioner group, patient population, indications, etc.
- Clinicians are frequently asked to undertake projects in their area of clinical expertise but they lack knowledge and skills in project management, implementation and evaluation.
- Clinicians are usually required to conduct a project in addition to their normal duties but without additional time or resources.
- Health service staff are well aware of their limitations and those of their colleagues in undertaking projects and they welcome advice and support.

### What factors influenced the decisions, processes and outcomes?

#### Factors that influenced decision-making for development of the support services

Each support service can be described with three main components (Fig. 4). The components were developed to meet the pilot objectives, overcome or minimise barriers and build on the enablers identified locally and from the literature, and address specific requests for content and format from the needs analysis.

Each support service was based on a solid foundation of research evidence and local data. The barriers, enablers and needs related to achieving the objectives are mapped to the relevant components of each intervention in Additional file 1. The findings of the local needs analysis are consistent with the current literature on EBDM using research and/or data [8–12, 15, 52, 62, 65, 91, 92], disinvestment and resource allocation [56–59, 61, 64, 70, 76, 93–95], and information needs of health service decision-makers [39, 40, 43, 45, 53, 89]. More recently, systematic reviews have identified interventions that have been demonstrated to enhance uptake of research evidence and these are also included in the matrix [8, 67, 96–99]. Two systematic reviews of interventions to improve use of data for health service decisions [100] and clinical decisions [101] were unable to find evidence of effective strategies.

Some barriers can be ameliorated but not removed entirely. For example, lack of time is a major issue. Education and capacity building may result in staff being more skilled and confident, and therefore quicker, at certain tasks which will reduce the problem of lack of time to some degree; but there may still be insufficient time to access and appraise evidence adequately. Providing additional staff time for data collection and data entry will assist staff delivering projects but, unless there are major changes in the health service environment, they will still have to undertake other project tasks in addition to their clinical duties and rely on their colleagues for informal backup.

There were other barriers which could not be addressed; for example those beyond the scope of the project, such as lack of computer access for nurses, or those outside the jurisdiction of the health service, such as variability in cost-accounting between institutions preventing cost comparisons.

#### Factors that influenced processes and outcomes of piloting the support services

The Capacity Building and Project Support Services were successful in achieving their short term objectives; but it is not known if workshop participants changed their practice to use the new knowledge and skills or if the disinvestment pilot project was fully implemented and evaluated appropriately. The Data Service was not implemented at all.

The factors influencing these outcomes are collated in Additional file 1 using the framework and taxonomy for evaluation and explication of evidence-based innovations [1]. However key factors for success and failure can be summarised very simply. Success was achieved when funding was available; activities were under the ownership of and within the expertise of the CCE project team; and where CCE or the SHARE Steering Committee had authority to implement change. Failure occurred in the absence of any one of these factors. Incorrect assumptions, inadequate barrier analysis and unforeseen events also played a part.

*Funding*

The SHARE Program was adequately resourced with funding from the DHS and Monash Health. However when the funding was reduced in the final year of the program the remaining implementation and evaluation activities were not undertaken.

*Ownership, expertise and authority*

One of the implementation strategies for the overall SHARE Program was to integrate the activities into the Monash Health Strategic and Business Plans and CCE was responsible for delivering them [6]. Theoretically this gave CCE ownership of the process and authority to implement most of the changes; changes beyond this remit could be authorised by members of the Steering Committee within their portfolios. The activities of the Evidence, Capacity Building and Project Support Services were all to be undertaken by CCE staff who were skilled and experienced in these areas and, if they were to be maintained beyond the SHARE Program, CCE would be the appropriate home for them. While the funding was available, these were delivered successfully.

However the activities of the Data Service were beyond the skill set of the CCE team and might be more appropriately delivered by the Clinical Information Management unit. Because these activities were outside the experience and expertise of CCE staff, a number of incorrect assumptions were made. It was assumed that data could be accessed as readily as research evidence and that data analysts would also have similar knowledge brokering skills to CCE staff. The Data Service proposal had not been discussed with the CIM Director, but with his Executive Director. In hindsight, it is clear that a proposal requiring such high level expertise should have been discussed with the technical expert as well as, or instead of, a strategic decision-maker. The CIM Director was as helpful as he could be, but the SHARE objectives were not within his work plan, other priorities were competing for his time, and there was nothing he could do about the lack of access and coordination of available datasets or the lack of capacity and capability to deliver the objectives.

Lack of ownership by key stakeholders [61, 102, 103] and lack of authority to make the proposed changes [10, 12, 61, 104–106] are well-recognised barriers to effective implementation.

*Barrier and enabler analysis for implementation strategies*

The SHARE team ascertained and analysed barriers, enablers and needs for use of evidence from research and data in decision-making and getting projects implemented and evaluated effectively (Additional file 1). The components of the support services were the interventions to address these issues. However less attention was given to additional barriers and enablers for implementation of these interventions. Two examples where this affected outcomes are under-utilisation of Capacity Building Service workshops and support sessions and inability to access all Monash Health datasets. The workshops and support sessions were designed to meet local needs for education, training and support; managers supported staff member’s participation; preferred formats were implemented; and participants found them valuable. Hence other factors are likely to have caused the poor attendance, such as issues with the venues or scheduled times, which may have been averted if known beforehand. Lack of coordination of health data is now well documented in the literature [62, 65, 107], but barriers to accessing data were not explored at the time of the pilot, contributing to development of the initial unrealistic proposal for the Data Service.

*Unforeseen events*

The unforeseen announcement of extension of the existing data warehouse had adverse impacts on the proposal for the Data Service being considered at the time.

The external factors that affected acceptance of pilot projects could also not have been anticipated but significantly limited implementation and investigation of the Project Support Service.

Potential withdrawal of health department funding in later stages of long term projects was a recognised risk. This was anticipated and discussed with the department while there was enough time to revise the proposed activities and funding was assured at this time. Several months later this decision was reversed. Resources that could have been used to evaluate earlier activities had been directed to implementation of additional activities; with the result that evaluation was significantly limited across all areas.

### Limitations

The findings come from one organisation and there may be many differences with other health services which limit generalisability. The level of expertise within the Centre for Clinical Effectiveness is unusual in this context. Although hospital-based resources for evidence synthesis are becoming more common [108, 109], they are not widespread, and the additional skills in implementation and evaluation are less common. Monash Health also had considerable capacity within the Clinical Information Management unit; the team of 12 skilled data analysts is larger than many local health services. Changes may be even more difficult in health services that do not have these resources.

The shortened timelines prevented implementation and evaluation of some activities. The small numbers of participants in the pilot processes and evaluations present similar weaknesses. Both limit the ability to draw firm conclusions from the findings.

### Implications for research, policy and practice

It is well documented that health service staff need education, training, support and assistance from experts to enable EBP; and effectiveness of evidence products and capacity building strategies to address this have been reported [3, 4, 8–13]. In-house ‘resource centres’ have been proposed as a solution [17, 59, 70–72] but, other than capacity building for research [88], we were unable to find any examples that had been evaluated. Monash Health had the expertise within CCE and CIM to provide assistance in all areas except health economics; however outside the SHARE funding, provision of assistance was curtailed by limited resources.

The Capacity Building and Project Support Services achieved their short term objectives and were well accepted. Incorrect assumptions and a series of unfortunately timed events prevented successful implementation of the Data Service during the SHARE timeframe; however the expanded data warehouse with improved access to a greater number of datasets increases the feasibility of this concept. All of the options considered still have potential to improve decision-making and project implementation and evaluation. Further exploration of support services is warranted.

The case studies presented here complement the existing disinvestment literature by providing details of local influencing factors and demonstrating their impact. This information may enable health service staff and researchers wishing to establish similar services to build upon the enablers and avoid or minimise the effect of the barriers.

Projects have costs, either in direct funds to pay for project staff or in lost opportunity costs for staff who cannot undertake clinical duties while engaged in project activities. If these projects are underpinned by incorrect non-evidence-based decisions, are not implemented effectively, or the evaluation findings are invalid or non-existent, the resources used will have been wasted. Based on theoretical evidence, support services should improve the quality of decisions, increase the success and sustainability of project objectives, and produce more trustworthy evaluations. Further research into the effectiveness and cost-effectiveness of support services is required.

Project funds are often insecure and evaluation is frequently the major casualty when funding ends prematurely [110]. When change is implemented, but not evaluated, it is not known whether the funding was used wisely or was a waste of money. This is ironic in investigation of disinvestment as the process does not meet the *“goal of effective use of scarce health care resources”* [17]. Project managers may wish to consider scheduling evaluation activities as early as possible to minimise the impact of loss of funds later in the project.

In their systematic review of information needs and information-seeking behaviour, Clarke and colleagues note the need for further investigation of the differences between health professional groups [39]. The differences between medical, nursing, allied health, management and support groups in our study may inform others researching in this area.

## Conclusion

Health service staff need access to education, training, expertise and support to enable evidence-based decision-making and to implement and evaluate the changes arising from those decisions. Three support services were proposed based on research evidence and local findings. Local factors, some unanticipated and some unavoidable, were the main barriers to successful implementation. All three proposed support services hold promise as facilitators of EBP in the local healthcare setting. The findings from this study will inform further exploration.

## Additional file

Additional file 1: Methods and Results. (PDF 1392 kb)

## Abbreviations

CCE: Centre for Clinical Effectiveness
CIM: Clinical Information Management
DHS: Department of Human Services
EBDM: Evidence-Based Decision-Making
EBP: Evidence Based Practice
ICD-10: International Statistical Classification of Diseases and Related Health Problems - 10th revision
RE-AIM: Reach, Effectiveness, Advocacy, Implementation, Maintenance
SHARE: Sustainability in Health care by Allocating Resources Effectively
TCP: Technology or clinical practice
TCPC: Technology/Clinical Practice Committee
UCSF: University of California San Francisco
VPACT: Victorian Policy Advisory Committee on Technology
