# Supplementary material for: Sustainability in Health care by Allocating Resources Effectively (SHARE) 7: supporting staff in evidence-based decision-making, implementation and evaluation in a local healthcare setting
Source: BMC Health Serv Res. 2017 Jun 21;17:430. doi: 10.1186/s12913-017-2388-8 (PMC5480160; doi:10.1186/s12913-017-2388-8)
Supplement: Additional file 1: — Methods and Results. (PDF 1392 kb) [file 12913_2017_2388_MOESM1_ESM.pdf]

The SHARE Program (Sustainability in Health care by Allocating Resources Effectively) 7: Supporting staff in evidence-based decision-making, implementation and evaluation in a local healthcare setting

Additional File: Methods and Results

Contents

DATA COLLECTION: Methods and Sources 2
NEEDS ANALYSIS: Survey of senior health service decision-makers 5
NEEDS ANALYSIS: Survey of pharmacists and members of pharmacy-related committees 12
NEEDS ANALYSIS: Survey of staff enrolling in an Evidence Dissemination Service 14
NEEDS ANALYSIS: Interviews with program, department and unit heads 16
NEEDS ANALYSIS: Differences between professional groups 19
DATA SERVICE: Interviews with staff responsible for collecting, maintaining and sharing data 20
CAPACITY BUILDING SERVICE: Evaluation Framework 24
CAPACITY BUILDING SERVICE: Evaluation Results 28
PROJECT SUPPORT SERVICE: Factors influencing the SHARE pilot disinvestment project 37
FACTORS THAT INFLUENCED DECISION-MAKING FOR SUPPORT SERVICES 38
FACTORS THAT INFLUENCED PROCESSES AND OUTCOMES OF SUPPORT SERVICES 44
REFERENCES 45

DATA COLLECTION: Methods and Sources

LITERATURE REVIEW

Information needs of decision-makers

Aim: To identify the information needs of decision-makers in local healthcare services to facilitate development of pilot support services

Questions: What are the information needs of clinicians and managers to support evidence-based decision making regarding the introduction or removal of technologies and clinical procedures?

How have assessments to determine these needs been conducted in the past?

Sources: Medline, CINAHL, EMBASE, LISA, LISTA and Google

Medline Search (adapted for other databases): (exp Needs Assessment/) AND (Information Dissemination/ or Information Services/ or Information Management/) limit to (English language and humans)
Google Search: (information OR evidence) AND (need OR assessment) AND (health OR nurs OR doctor OR med). Preferences were set to English language

Inclusion criteria: Articles describing information needs assessments in similar health service contexts examining how clinicians and managers make evidence-based decisions regarding the introduction or removal of technologies and clinical practices; articles published in English from 1996

Exclusion criteria: Information needs of students; continuing professional education needs; point of care decision-making needs; assessments of information needs in resource poor health settings

Data Collection and Analysis: Inclusion, exclusion and appraisal criteria were established a priori. Studies to be reviewed by one reviewer in consultation with colleagues when necessary. Critical appraisal relevant to study design to be conducted using standard CCE templates.

Results: No studies were found to meet the inclusion criteria. The limitations of the very specific question and narrow selection criteria were acknowledged. Earlier broad searches resulted in unmanageable numbers of returned articles, however limiting the search returned none. Since the purpose of the review was to inform development of the support services, and not to be a systematic review providing a definitive answer for others, a decision was made to take a pragmatic, iterative approach by accessing relevant publications already known to the project team and following up with simpler searches, pursuing articles from reference lists, etc.

SURVEYS

Staff who made decisions about resource allocation

Aim: To identify the information needs of decision-makers at Monash Health to facilitate development of support services and gather baseline data for evaluation purposes

Participants: Staff who made decisions regarding resource allocation for technologies and clinical practices

Design and content: An electronic questionnaire was designed and delivered using SurveyMonkey [1]. Questions were developed to identify current use of evidence; confidence in searching for, accessing and appraising evidence; difficulties in using evidence and implementing evidence-based change; preferred content and format of bulletins disseminating research evidence; and preferred formats for education and training in these areas. Some questions were adapted from Taylor et al [2].

Pretesting and piloting: The survey was pre-tested with colleagues at a co-located research institute, piloted with the SHARE Steering Committee, and refined based on feedback from these groups

Distribution: An email with an embedded link to the survey was distributed to senior staff using the Monash Health 'All Managers' and 'Senior Medical Staff' email lists. Members of these lists were asked to forward the survey to others who made decisions about resource allocation but might not be on the list.

Data collection: Data were collected over a four week period from the time of distribution. No reminders were sent.

Analysis: Results were downloaded into Excel from the survey provider. Qualitative data from the three free text answers were copied into NVivo [3] where they were coded according to themes presented in Michie et al [4]. Data were reviewed by two investigators to ensure agreement of coding. Discrepancies were discussed with a third investigator until consensus was reached. There were insufficient categories in the Michie et al framework to address some of the organisational issues; additional sub-themes were created as required.

Response rate: 141 staff members responded. 118 were eligible to complete the survey having answered 'yes' to the screening question asking if they made decisions about resource allocation. 103 completed the entire survey. The response rate could not be calculated in the absence of denominator information; the total number of staff on the email lists and the number of additional staff to whom the survey was forwarded were unknown.

Representativeness of sample: All programs and service sites were represented in proportions consistent with the size of the program or campus. A range of professional disciplines were represented: nursing (28%), allied health (25%), medical (24%) and other (23%) including pharmacy, diagnostic services, corporate and clinical program management, and administration.

Pharmacists and members of pharmacy-related committees

Aim: To identify pharmacists and members of pharmacy-related committees who make, implement and/or evaluate decisions regarding pharmaceuticals and related equipment; identify those who would like to receive training in evidence-based practice change; and their preferred formats for training

Participants: Pharmacy staff and members of pharmacy-related committees (Therapeutics, Medication Safety and Adverse Drug Reaction Committees; High Cost Drugs Working Party)

Design and content: An electronic questionnaire was designed and delivered using SurveyMonkey [1]. Questions were developed to identify preferred formats for education and training and factors that would facilitate participation.

Distribution: An initial invitation and a reminder invitation were sent one week apart. An initial mailing list of 214 Southern Health staff members was established. Email addresses were obtained from the Pharmacy secretary, the internet and, where not available through these means, based on the standard Southern Health email format of firstname.lastname@southernhealth.org.au.

Data collection: The survey was open for 12 days.

Analysis: Results were downloaded into Excel from the survey provider. Qualitative data from the three free text answers were copied into Nvivo [3] where they were coded according to themes presented in Michie et al [4]. Data were reviewed by two investigators to ensure agreement of coding. Discrepancies were discussed with a third investigator until consensus was reached. There were insufficient categories in the framework by Michie et al to address some of the organisational issues; additional sub-themes were created when required.

Response rate: 60 staff members responded to the survey for a response rate of 34% (60/177). 37 email addresses bounced or addressees were on leave when the first email was sent. The reminder email was sent to 177 staff and 2 additional staff members were on leave. 177 staff received one or both emails inviting them to participate in the survey.

Representativeness of sample: A broad range of pharmacist's roles (including management, clinical and technical responsibilities) and all committees were represented.

Staff enrolling in an Evidence Dissemination Service

Aim: To ascertain how participants enrolling in an Evidence Dissemination Service (EDS) currently use evidence in decision-making

Participants: Staff members enrolling to participate in EDS

Design and content: An electronic questionnaire was designed and delivered using SurveyMonkey [1]. Questions were developed to identify current use of evidence; time spent in searching for, accessing and appraising evidence; perceptions of EBP at Monash Health and features of respondent's decision-making practice.

Distribution and Data collection: The survey was part of the enrolment process.

Analysis: Results were downloaded into Excel from the survey provider

Response rate: 46 staff members enrolled to participate in EDS during the survey period.

Representativeness of sample: Respondents represented all clinical groups and all health service programs and sites.

INTERVIEWS

Members of organisation-wide committees, representatives of approved purchasing units and individuals who made decisions about resource allocation

Aim: 1) To examine and document current processes for making, implementing and evaluating decisions and the factors that influence them (all interviewees) and 2) To identify relevant issues and pilot draft questions for needs analysis survey (clinical program managers only)

Participants: Invitations were extended to 1) representatives of 14 committees with a mandate to make organisation-wide decisions regarding allocation of resources, 2) managers of 5 approved purchasing units (APUs) and 3) 9 managers from one clinical program selected for its high use of health technologies.

Interview schedule: Questions were designed to identify how evidence and data were used in decision-making, implementation and evaluation and the associated barriers and enablers (all interviewees). Additional questions were designed to identify training and support needs for decision-making, implementation and evaluation and preferred formats for delivery (clinical program managers only). These were part of a schedule investigating organisational decision-making more broadly. The full interview schedule is available [5].

Data collection: Interviews were approximately 1 hour long. Two CCE staff members attended, one as facilitator, one as note taker. Drafts were sent to the interviewees for clarification, comment and/or amendment as required.

Analysis: Final interview notes were analysed thematically in MS Word and Excel using the elements of the theoretical framework.

Response rate: 13 of the 14 committees, all 5 APU managers and all 9 clinical managers participated

Representativeness of sample: All but one of the relevant committees and all APUs were represented, the clinical managers selected represented Program Directors, Medical Department Heads, Nurse Unit Managers and Quality and Risk Manager in medical and surgical sub-specialties, nursing and quality management across a range of campuses.

**Staff who had undertaken projects implementing disinvestment-type activities**<br>**Aim:** To learn from previous experiences at Monash Health<br>**Participants:** Invitations were extended to project managers of 10 projects that had undertaken disinvestment-type activities ie removal, restriction or replacement of TCPs in current use. Projects were identified during the interviews with decision-makers noted above followed by a snowballing exercise with managers of projects identified.<br>**Interview schedule:** Questions were designed to explore how routinely-collected hospital data, other local data and research evidence were used in the development and implementation of projects; barriers and enablers to successful project implementation; what staff would do again and what they would do differently [5]<br>**Data collection:** Interviews were approximately 1 hour long. Two CCE staff members attended, one as facilitator, one as note taker. Drafts were sent to the interviewees for clarification, comment and/or amendment as required.<br>**Analysis:** Final interview notes were analysed thematically in MS Word and Excel according to the theoretical framework adapted for SHARE [6]<br>**Response rate:** Representatives of all 10 projects participated<br>**Representativeness of sample:** The process was designed to be illustrative rather than comprehensive. A range of project topics were included, no attempt was made to ascertain all possible projects.
**Representatives from departments collecting, maintaining and sharing data related to TCPs**<br>**Aim:** To identify current sources of data at Monash Health and the processes involved<br>**Participants:** Departments were identified by the Head of Clinical Information Management, a concept paper on knowledge transfer at Monash Health and via a snowballing technique asking respondents if they were aware of others. Representatives of 10 relevant departments were invited to participate (Clinical Information Management, Health Information Systems, Pharmacy, Pathology, Diagnostic Imaging, Research Directorate, Infection Control, Infectious Diseases and the Clinical Audit and Clinical Risk groups within the Quality Unit).<br>**Interview schedule:** Questions were designed to identify the data available, methods of collection and storage, utilisation in decision-making, internal and external reporting, other forms of dissemination, strengths and weaknesses of the current system and opportunities for improvement.<br>**Data collection:** Interviews approximately 1 hour long were conducted by one CCE staff member and audio taped where possible.<br>**Analysis:** Interview data were collated in Excel and analysed thematically in Nvivo [3].<br>**Response rate:** All 10 invitees participated<br>**Representativeness of sample:** A broad range of settings were included.

NEEDS ANALYSIS: Survey of senior health service decision-makers

Note: percentages may not add to 100 due to rounding.

DEMOGRAPHICS

Question 1: Does your role at Southern Health involve decision making about introducing or changing use of technologies or clinical practices?

Yes	118
No	23
Total	141

The 23 respondents who answered 'No' to this first question were not asked any further questions and thus response rates to subsequent questions are calculated based on 118 respondents.

Question 2: What is your role at Southern Health?

Role	n (%)
Nursing	33 (28.0)
Medical	28 (23.7)
Allied Health	30 (25.4)
Other	27 (22.9)

Respondents describing their role as 'Allied Health' or 'Other' were asked for further detail; 36 of the 57 respondents provided further information. There was variation in how this information was provided with some people providing explicit job titles, such as Director of a specified service and others providing a work area, such as physiotherapy.

Allied health	Other
▪ Physiotherapists (n=6)	▪ Program or project management (n=7)
▪ Occupational therapists (n=5)	▪ Director or service coordinator, administration (n=3 each)
▪ Speech pathologists, Podiatry (n=2 each)	▪ Midwifery, medical scientist (n=2 each)
▪ Director (n=1)	▪ Pharmacist, information technology, psychology (n=1 each)

Question 3: In which Program do you work?

Program	n	%
Continuing Care	21	17.8
Corporate Office	5	4.2
Medicine Program	16	13.6
Mental Health Program	11	9.3
Shared Services	7	5.9
Specialty Program	9	7.6
Strategy, Performance and Planning	3	2.5
Surgery Program	6	5.1
Women's and Children's Program	15	12.7
Other	25	21.2

Those responding as 'Shared Services' or 'Other' were asked for further detail; 27 of the 32 respondents provided further information

Shared services	Other
▪ Pharmacy, Information Technology (n=1 each)	▪ Southern Metropolitan Integrated Cancer Services (n=4)
▪ Pathology, Diagnostic Imaging, Site Management (n=2 each)	▪ Acute program (n=3)
	▪ Community Health, acute ambulatory (n=2 each)
	▪ Critical care, anaesthetics, community rehabilitation (n=1 each)
	▪ Physiotherapy (n=4)
	▪ Dietetics, podiatry (n=1 each)

Question 4: At which Southern Health sites do you work?

It was recognised in piloting that some staff consider that they work equally at more than one campus and did not identify a 'main' site of work. Alternatively they may have considered one site their main clinical site and another site their main administrative site. Thus respondents could select more than one site of work and the 118 respondents gave 181 answers to this question.

Site	n	%
Casey	23	12.7
Clayton	71	39.2
Cranbourne	7	3.9
Dandenong	39	21.5
Kingston	15	8.3
Moorabbin	18	9.9
Other	8	4.4

Other sites included: Community Mental Health Sites, Jessie McPherson, Berwick Community Health Service (CHS), Pakenham CHS and Dandenong CHS.

Summary of demographic data

We are confident that the respondents represent the range of Programs and roles at Southern Health and that the percentage of respondents from different sites is representative of the staff numbers at different sites. Initially it was proposed that we would have a second round of survey distribution, targeting areas, sites or Programs that were not appropriately represented however due to the adequate response rate to initial survey distribution this was not considered necessary.

CURRENT USE OF EVIDENCE

Several questions were asked to examine the respondent's current use of evidence, the type of evidence they use and their confidence in finding and appraising evidence. There are two purposes to these questions:

- It may be possible to survey this population again at a later date and see if there is any shift in self-reported use of and confidence in using evidence.
- Reports of confidence in finding and appraising evidence will inform development of a capacity building service by giving an initial indication of areas of course demand.

Question 5: In your decision making around introduction of or change in use if technologies or clinical practices, approximately how often do you include evidence from research?

Frequency	n	%
Never	0	0.0
Rarely	9	7.6
Sometimes	24	20.3
Often	47	39.8
Always	36	30.5
Did not answer	2	1.7

Although only 30% of all respondents reported 'Always' using evidence in decision-making, 57% of respondents in a Medical role, 15% Nursing, 20% Allied Health and 36% Other reported 'Always' using evidence.

Question 6: How often do you use the following sources of information to make decisions about technologies or clinical practices?

Source	Never n (%)	Rarely n (%)	Sometimes n (%)	Often n (%)	Always n (%)	Often or Always	Total
Colleagues	0 (0.0)	3 (2.7)	21 (19.1)	58 (52.7)	28 (25.5)	86 (78.2)	110
Clinical Practice Guidelines	2 (1.8)	8 (7.2)	25 (22.5)	53 (47.7)	23 (20.7)	76 (68.5)	111
Original research	2 (1.8)	23 (20.7)	31 (27.9)	40 (36.0)	15 (13.5)	55 (46.6)	111
Systematic reviews	8 (7.2)	28 (25.2)	24 (21.6)	38 (34.2)	13 (11.7)	51 (45.9)	111
Textbooks	11 (9.9)	25 (22.5)	42 (37.8)	26 (23.4)	7 (6.3)	33 (29.7)	111

These were also analysed by professional role. While there were no major differences by role in the use of colleagues and clinical practice guidelines, the use of systematic reviews 'often' or 'always' varies from 71% in Medical staff, 47% Allied Health, 37% Others to just 21% in Nursing. Similar rates were found in the use of original research with 75% of Medical staff using it 'often' or 'always', and only 37% and 38% for Nursing and Allied Health respectively. People from Other (clinical support, shared services) roles used original research 'often' or 'always' 50% of the time.

Sixteen (13%) people identified 'Other' sources of information used to make decisions about technologies or clinical practices. These sources included the professional database 'Up to date' (n=3) and one mention each of:

- Professional Organisations ACC, ECS, AHA

- Consumers
- Society for Simulation in Healthcare listserve
- Professional association meetings
- Clinical meetings, scientific meetings, workshops
- Members of international multicentre trial groups
- Literature review from Centre for Clinical Effectiveness
- Other institutions
- Industry guidelines and specifications
- Clinical experts
- Studies in change management, supervision, panel discussions
- Mentoring in-house education portfolio responsibility
- Chair of Nursing Research team

Question 7: How often do you use the following resources to find information about technologies or clinical practices?

Resource	Never n (%)	Rarely n (%)	Sometimes n (%)	Often n (%)	Always n (%)	Often or Always	Total
Internet search engines (eg Google)	4 (3.6)	9 (8.1)	36 (32.4)	47 (42.3)	15 (13.5)	62 (55.9)	111
Electronic databases (eg Medline)	10 (9.0)	12 (10.8)	32 (28.8)	46 (41.4)	11 (9.9)	57 (51.4)	111
Guidelines websites	11 (10.0)	12 (10.9)	36 (32.7)	42 (38.2)	9 (8.2)	51 (46.4)	110
Personal subscriptions to journals	22 (19.8)	15 (13.5)	33 (29.7)	33 (29.7)	8 (7.2)	41 (36.9)	111
The Cochrane Library	24 (21.6)	29 (26.1)	28 (25.2)	27 (24.3)	3 (2.7)	30 (27.0)	111
Personal subscriptions to email listserves	31 (28.2)	25 (22.7)	27 (24.5)	21 (19.1)	6 (5.5)	27 (24.5)	110
Library hard copy journals	19 (17.3)	38 (34.5)	41 (37.3)	11 (10.0)	1 (0.9)	12 (10.9)	110

The most frequently used resource for finding information is the internet with 55.9 percent of respondents often or always using search engines such as Google. Also highly used are electronic databases such as Medline (51.4%) and guidelines websites (46.4%). Though the previous question showed that 45.9 percent of respondents used systematic reviews for decision making only 27 percent of respondents always or often used the Cochrane Library.

Cochrane Library	Never or rarely n (%)	Sometimes n (%)	Often or always n (%)	Total
Nursing	18 (55)	9 (22)	5 (15)	32
Medical	9 (32)	8 (28)	11 (39)	28
Allied Health	13 (43)	7 (29)	9 (30)	29
Other	13 (48)	4 (18)	5 (19)	22
Total	53 (48)	28 (25)	30 (27)	111

Clear professional differences were observed at this level with more than half (55%) of Nursing respondents 'rarely' or 'never' using the Cochrane library, while more than two-thirds (67%) of Medical staff report using it 'sometimes', 'often' or 'always'. However, resources such as Medline and PubMed also identify systematic reviews.

Sixty-five percent of Medical staff use other electronic databases of research 'often' or 'always' versus only 34.4 percent of Nursing staff.

Thirteen people (11% of respondents) identified other resources for finding information. Other included:

- Library e-journals (n = 2)
- Conferences (n = 2)
- Professional association meetings
- Clinical meetings, scientific meetings, workshops
- Through web-based updates to all current clinical trials on which our patients are enrolled
- Up to date
- Information from other professional bodies involved in the same program type
- Drug company trial information
- Guidelines from professional bodies
- Research Centres - Heart
- Company with technology

Again, the small number of respondents answering 'Other' means it is difficult to conclude anything about these sources and how important they are to the decision makers. It should be noted that information direct from the company may contain a bias and it is unknown how many professional association meetings, and other meetings and workshops are sponsored by pharmaceutical or medical equipment companies.

Question 8: How confident are you in the following:

	Very or Quite confident n (%)	Very confident n (%)	Quite confident n (%)	Moderately confident n (%)	Not very confident n (%)	Not at all confident n (%)	Don't know n (%)	Total n
Searching for evidence (eg using Medline to conduct a search)	57 (51.8)	22 (20.0)	35 (31.8)	29 (26.4)	15 (13.6)	6 (5.5)	3 (2.7)	110
Accessing the evidence (eg finding a full text copy of the article)	55 (50.0)	25 (22.7)	30 (27.3)	33 (30.0)	15 (13.6)	6 (5.5)	1 (0.9)	110
Using the evidence to make decisions	51 (46.4)	13 (11.8)	38 (34.5)	41 (37.3)	16 (14.5)	2 (1.8)	0 (0.0)	110
Implementing changes based on the evidence	50 (45.5)	13 (11.8)	37 (33.6)	44 (40.0)	12 (10.9)	4 (3.6)	0 (0.0)	110
Appraising the evidence (eg assessing the quality of the study)	37 (33.6)	9 (8.2)	28 (25.5)	39 (35.5)	26 (23.6)	7 (6.4)	1 (0.9)	110

Whilst the level of any confidence (moderately, quite or very confident) regarding the searching for, accessing, appraising and using evidence and implementing changes based on the evidence is relatively high (up to 85%) the proportion of people with higher levels of confidence (very or quite confident) drops to approximately 50 percent, and the proportion who are very confident is around 22 percent and lower. Only 34 percent of respondents were very or quite confident at appraising the evidence and 30 percent had little or no confidence at appraising the evidence. These confidence scores represent obvious opportunities for training and knowledge improvement.

There appears to be role differences in searching for evidence with 46.4 percent of Medical staff feeling 'very confident', but only about 10 percent in Nursing and Allied Health (9.7%, 10.3% respectively). Similar proportionate differences remain when "very confident' responses are combined with 'quite confident', and persist in all remaining option responses.

Question 9: How confident are you at assessing these aspects of a published paper?

	Very or Quite confident n (%)	Very confident n (%)	Quite confident n (%)	Moderately confident n (%)	Not very confident n (%)	Not at all confident n (%)	Don't know n (%)	Total n
Relevance to your situation	50 (45.9)	17 (15.6)	33 (30.3)	47 (43.1)	10 (9.2)	0 (0.0)	2 (1.8)	109
Study design	34 (31.2)	14 (12.8)	20 (18.3)	35 (32.1)	31 (28.4)	5 (4.6)	4 (3.7)	109
Influence of bias	30 (27.5)	8 (7.3)	22 (20.2)	32 (29.4)	40 (36.7)	3 (2.8)	4 (3.7)	109
Adequacy of sample size	26 (23.9)	10 (9.2)	16 (14.7)	38 (34.9)	34 (31.2)	7 (6.4)	4 (3.7)	109
Trustworthiness of an article	24 (22.0)	6 (5.5)	16 (14.7)	47 (43.1)	32 (29.4)	5 (4.6)	3 (2.8)	109
Statistical tests / principles	15 (13.8)	8 (7.3)	7 (6.4)	27 (24.8)	49 (45.0)	15 (13.8)	3 (2.8)	109

Whilst 34 percent of respondents to question nine were confident in appraising the evidence, when asked about their confidence at assessing specific aspects of quality the confidence levels drop. Though 46 percent of respondents are very or quite confident at assessing the relevance of an article to their situation, only 14 percent are equally confident at assessing the statistical tests and principles applied in the article. Overall, only 22 percent are very or quite confident at assessing the overall trustworthiness of an article.

After combining responses for 'quite' and 'very confident', Medical staff report significantly higher levels of confidence for all aspects of assessing a published paper.

Again, as with the results for question nine, these results indicate a clear opportunity for further training to improve knowledge and confidence.

DIFFICULTIES WITH USING EVIDENCE AND IMPLEMENTING EVIDENCE-BASED CHANGE

Respondents were invited to reply to the following two open ended statements and responses to these statements were analysed thematically. However, each theme is not mutually exclusive.

Question 10: Please describe any difficulties you have with searching for, accessing and appraising evidence from the research literature and using this evidence in decision making around technologies and clinical practices.

Sixty-one people answered this open-ended question.

While the question was specifically aimed at problems people encountered searching, accessing, appraising, and using research literature, themes arose around more generic issues with incorporating research into everyday practice and decision making. These are:

- Environmental context and resources; including information inaccessibility, person and their environment, material resources (availability and management), and time (n = 42)
- Skills (n = 26)
- Knowledge; including gaps in evidence (n = 14)
- Social influences; difficulties accessing support, and conflicting/ competing demands (n = 4)
- Professional role, identity and boundaries (n = 4)
- Beliefs about capabilities; professional and self-confidence (n = 3)
- Emotion; mainly fear (n = 1)

"The difficulty lies in the time required to do a comprehensive literature review. My other concern is accessing help and advice in writing up a literature review. When I have requested help in this area before, I have been told that we don't have the resources to assist me with this. I would like to improve my skills in writing literature reviews and without being tied to a research degree through a university there doesn't seem to be obvious support for a clinician such as myself."

"Time. No time allocated to access, review or to develop an evidence-based model to implement into practice."

"Difficulty understanding the trustworthiness of what I am reading as I do not understand the statistics and limited knowledge of bias."

"So much information, not sure how to narrow it. Worried that I've missed an important piece of information that might completely change the decision making process."

Question 11: Please describe any difficulties you have with implementing evidence-based practice changes.

Fifty-nine people answered this open-ended question.

Respondents identified a number of common change management problems, as well as some specific to Southern Health. These are (from most cited to least):

- Social influences; further divided into organisational culture/climate, conflict/competing demands, organisational structure, change management, team working, lack of support, negotiation, organisational commitment/alienation, power/hierarchy (n = 49)
- Environmental context and resources; including information inaccessibility, person and their environment, material resources (availability and management), and time (n =31)
- Emotion; resistance to change, and negative affect (n =10)
- Skills (n = 7)
- Knowledge; including gaps in evidence (n =6)
- Behavioural regulation; including difficulties with feedback, implementation intention, moderators of intention-behaviour gap, and project management (n = 5)
- Professional role, identity and boundaries (n = 4)
- Nature of the behaviours (n = 4)
- Motivation and goals (n = 2)
- Beliefs about consequences (n =1)

"Things move very slowly - everyone is busy. Difficult to get others around to help with implementing change. We have good ideas, but getting everyone to work together on a project to achieve a real outcome is extremely difficult. Lots of different skill levels, and different level of priority placed on being evidence based. To do things properly takes time and most people just want to tick something off their list and cut corners - very frustrating."

"Staff resistance, budget, resources, the size of the organisation and difficulty in getting even minor changes approved. Knowing which evidence-based practice is the one to use."

"Cost of these changes and sourcing the necessary equipment. Time constraints, medical acceptance/culture - this is the way we do things."

"Southern Health is a very large organisation. Disseminating information and influencing culture change can be difficult. More resources need to be developed for communication strategies across the organisation. For example not all staff have access to email. Not all staff have access to computers. It is difficult to ensure the governance around decisions made because you cannot ensure ALL staff are aware of changes. Shift work, sick leave, part-time staff are difficult to get too. Some issues that affect multiple disciplines such as falls prevention are not adopted in some areas as easily as others."

EVIDENCE SERVICE

Southern Health is exploring the establishment of an evidence service that would disseminate new information about technologies and clinical practices. Questions were asked about what type of information clinicians and managers might like to receive and in what format.

Question 12: When making decisions about health care technologies or clinical practices, what research information would you like to have available? (Respondents were invited to choose as many as applied).

Information Type	n	%
Critical appraisals of primary research	88	83.0
Full text of secondary research (eg. evidence-based guidelines, systematic reviews)	83	78.3
Critical appraisals of secondary research	79	74.5
Full text of primary research (eg. clinical trials)	73	68.9
Abstracts of primary research	50	47.2
Abstracts of secondary research	44	41.5
Other	7	6.6
Total response	106	100.00

Other included: consumer perspectives, case-studies of other health services, web-access to journals, professional guidelines and web-access for participation in group wide trials

Question 13: What focus would you prefer the research information you receive to have? (Respondents were asked to rank at least three preferences with 1 being the most preferred option.)

	1<br>n (%)	2<br>n (%)	3<br>n (%)	4<br>n (%)	5<br>n (%)	6<br>n (%)
Condition specific information (eg. Diabetes)	25 (23.8)	26 (25.2)	18 (17.5)	7 (13.0)	8 (20.0)	3 (21.4)
Professional group information (eg Emergency Department Nursing)	23 (21.9)	25 (24.3)	17 (16.5)	8 (14.8)	6 (15.0)	0 (0.0)
Program relevant information (eg. Mental Health)	21 (20.0)	20 (19.4)	26 (25.2)	16 (29.6)	2 (5.0)	0 (0.0)
Organisation wide information (eg. Infection Control)	15 (14.3)	14 (13.6)	15 (14.6)	14 (25.9)	16 (40.0)	1 (7.1)
Unit relevant information (eg. Monash Newborn Services)	13 (12.4)	18 (17.5)	26 (25.2)	9 (16.7)	8 (20.0)	2 (14.3)
Other	8 (7.6)	0 (0.0)	1 (0.97)	0 (0.0)	0 (0.0)	8 (57.1)
Total	105	103	103	54	40	14

Other Included:

- Consumer initiated, focused and developed research
- International relevance
- Focus needed depends on the task
- Skill or procedure specific e.g. bed management

Question 14: In what format would you prefer to receive information from research? (Respondents were asked to rank at least three preferences with 1 being the most preferred option.)

	1<br>n (%)	2<br>n (%)	3<br>n (%)	4<br>n (%)	5<br>n (%)	6<br>n (%)	7<br>n (%)
Short pdf attachment to an email (eg titles and hyperlinks)	33 (32.4)	19 (18.8)	26 (25.5)	5 (11.9)	0 (0.0)	0 (0.0)	0 (0.0)
Long pdf attachment to an email (eg titles, abstracts and hyperlinks)	26 (25.5)	22 (21.8)	11 (10.8)	8 (19.0)	2 (6.3)	3 (10.7)	0 (0.0)
Email with titles and embedded hyperlinks	18 (17.6)	26 (25.7)	21 (20.6)	2 (4.8)	7 (21.9)	4 (14.3)	0 (0.0)
Searchable database	18 (17.6)	13 (12.9)	19 (18.6)	12 (28.6)	6 (18.8)	7 (25.0)	1 (7.7)
Short paper-based newsletter (eg titles and web addresses)	4 (3.9)	14 (13.9)	13 (12.7)	9 (21.4)	6 (18.8)	5 (17.9)	1 (7.7)
Long paper-based newsletter (eg titles, abstracts and web addresses)	2 (1.9)	6 (5.9)	9 (8.9)	6 (14.3)	11 (34.4)	12 (42.3)	4 (30.8)
Other	1 (1.0)	1 (1)	3 (2.9)	0 (0.0)	0 (0.0)	0 (0.0)	7 (53.9)
Total	102	101	102	42	32	28	13

Based on first preferences the most preferred format for receiving information from research was as a short PDF attachment, followed by a long pdf attachment. An email with titles and hyperlinks and a searchable database were equally the third most preferred methods.

When first and second preferences were combined short pdf attachments, long pdf attachments and emails with embedded hyperlinks remain the most preferred options.

Other included:

- Short summaries about the article and main findings and then a link to the full article (like Medscape email alerts)
- Lectures and/or in-services
- Website
- Full text review articles by well-respected authors
- Workshops regarding methods eg. statistics, database development

EDUCATION AND TRAINING SERVICE

In addition to disseminating evidence, Southern Health, recognising that some staff may require assistance in making the best use of evidence, intends to provide support through education and training. Questions were asked about preferences regarding options for an education and training service.

Question 15: If we were to develop an education service to assist you in searching for, understanding and using research information to make decisions how would you prefer to participate? (Respondents were asked to rank as many options as they liked, with 1 being the most preferred option.)

	1<br>n (%)	2<br>n (%)	3<br>n (%)	4<br>n (%)	5<br>n (%)	6<br>n (%)
Self-paced online tutorials	36 (36.0)	11 (11.3)	14 (15.2)	10 (18.2)	15 (29.4)	3 (7.1)
Interactive workshops (eg ½ to 1 day)	21 (21.0)	24 (24.7)	9 (9.8)	10 (18.2)	6 (11.8)	0 (0.0)
Short courses (eg 1 to 3 days)	20 (20.0)	23 (23.7)	23 (25.0)	8 (14.5)	3 (5.9)	5 (11.9)
Lecture series (eg 1 hour per week for 10 weeks)	14 (14.0)	16 (16.5)	16 (17.4)	9 (16.4)	13 (25.5)	6 (14.3)
Journal club	6 (6.0)	11 (11.3)	8 (8.7)	11 (20.0)	8 (15.7)	16 (38.1)
Printed workbook	3 (3.0)	12 (12.4)	22 (23.9)	7 (12.7)	6 (11.8)	12 (28.6)
Total	100	97	92	55	51	42

Based on first preferences the most preferred form of education to aid in searching for, accessing and appraising evidence was a self-paced online tutorial, followed by an interactive workshop and then a short course.

When first and second preferences are combined self-paced online tutorials remain the preferred teaching method followed very closely by interactive workshops and then short courses.

When combining first and second preference by role, Medical staff prefer self-paced online tutorials, Nursing interactive workshops, and Allied Health short courses. Other staff hold a preference equally for lecture series and interactive workshops.

Question 16: If we were to develop a service to train you to implement evidence-based change, how would you prefer to receive this training? (Respondents were asked to rank as many options as they liked, with 1 being the most preferred option.)

	1<br>n (%)	2<br>n (%)	3<br>n (%)	4<br>n (%)	5<br>n (%)
Interactive workshops (eg ½ to 1 day)	35 (34.3)	23 (24.0)	15 (16.5)	7 (11.5)	4 (6.7)
Self-paced online tutorials	31 (30.4)	14 (14.6)	16 (17.6)	19 (31.1)	13 (21.7)
Short courses (eg 1 to 3 days)	23 (22.5)	25 (26.0)	19 (20.9)	11 (18.0)	6 (10.0)
Lecture series (eg 1 hour per week for 10 weeks)	9 (8.8)	19 (19.8)	25 (27.4)	8 (13.1)	18 (30.0)
Printed workbook	4 (3.9)	15 (15.6)	16 (17.6)	16 (26.2)	19 (31.7)
Total	102	96	91	61	60

Based on first preferences the most preferred was an interactive workshop, followed closely by self-paced online tutorial, then short courses.

When first and second preferences were combined the most preferred teaching method was interactive workshops, followed by short courses and then self-paced on-line tutorials.

Again, Medical staff have a preference – when combining first and second preferences – for self-paced online tutorials, however Nursing, Allied Health and Other all prefer interactive workshops, while Nursing staff equally prefer short courses.

Additionally, respondents were given the opportunity to suggest any other teaching methods that should be considered. Five people gave suggestions including; site-based mentoring, mentoring with clinicians of similar experience but in different disciplines, and a remote support service.

RESPONDENTS' ADDITIONAL THOUGHTS

Respondents were given the opportunity, through the open ended question 'Is there anything else you would like us to know?" to comment or provide additional information on any of the topics covered in the survey.

Fifteen people provided additional comments which were thematically analysed. The main themes to emerge are that:

- People want training and support
- The Southern Health website is difficult to use and it is hard to find relevant information
- Hard to find out if others in Southern Health have already done similar work – lack of resource/information sharing
- Evaluation of IT components is complex and time consuming

NEEDS ANALYSIS: Survey of pharmacists and members of pharmacy-related committees

At which Southern Health site do you work? (please select all that apply)

Site	Response
Clayton or Jessie McPherson	41
Casey or Cranbourne Integrated Care	6
Dandenong	16
Kingston	3
Moorabbin	5
Total respondents	60
Total responses	71

What is your role at Southern Health in relation to pharmaceuticals? (please select all that apply)

Role	Response
Adverse Drug Reaction Committee member	3
High Cost Drugs Working Party member	2
Medication Safety Committee member	7
Therapeutics Committee member	9
Clinical Drug Trials pharmacist	2
Clinical pharmacist	32
Outpatient / Discharge Services pharmacist	16
Pharmacy management	6
Pharmacy technician	2
Quality Use of Medicine and related areas pharmacist	6
Sterile Product Services pharmacist	7
Other	7
Total respondents	60
Total responses	93

Other included chairperson of research planning group (1), senior medical staff (1), clinician (1), therapeutic drug monitoring committee chairperson (1), compounding and drug distribution (1) and outreach pharmacist (2).

What is your role at Southern Health with relation to the pharmaceutical decision making process? (please select all that apply)

Role	Response
I source and appraise evidence to support change in practice	15
I am involved in making decisions regarding introduction or change in use of pharmaceuticals and related equipment	19
I implement practice change	22
I evaluate practice change	16
I do not currently do any of the above but expect to in the future	16
I do not currently do any of the above nor do I expect to do so in the future	8
Total respondents	58

Would you be interested in receiving training in any of these areas? (please select all that apply)

Topic	Response
Sourcing and appraising evidence to support changes in practice	35
Using evidence to inform decision making	45
Implementing practice change	43
Evaluating practice change	44
I am not interested in training in any of these areas at this time	4
Total respondents	58

Interestingly, although eight people felt they were not currently involved in the pharmaceutical decision making process nor did they expect to become involved in the future, four were still interested in some sort of training.

If we were to develop an education service how would you prefer to participate in training in the following areas:

Searching for, understanding and using information in decision making

Method	Combined 1st and 2nd	First preference	Second preference
Interactive workshops (eg ½ to 1 day)	24	9	15
Short course as a series (eg ½ day per week for 4 weeks)	22	7	15
Self-paced on line tutorials	21	14	7
Short course as a block (eg 2 days)	20	14	6
Lecture series (eg 1 hour per week for 10 weeks)	9	6	3
Printed workbook	7	2	5
Journal club	2	1	1

Implementation of evidence based practice change

Method	Combined 1st and 2nd	First preference	Second preference
Interactive workshops (eg ½ to 1 day)	29	16	13
Short course as a block (eg 2 days)	24	13	11
Short course as a series (eg ½ day per week for 4 weeks)	15	7	8
Self-paced on line tutorials	15	10	5
Printed workbook	9	3	6
Special Interest group	6	3	3
Lecture series (eg 1 hour per week for 10 weeks)	5	1	4

Evaluation of evidence based practice change

Method	Combined 1st and 2nd	First preference	Second preference
Interactive workshops (eg ½ to 1 day)	23	15	8
Short course as a block (eg 2 days)	23	11	12
Short course as a series (eg ½ day per week for 4 weeks)	18	7	11
Self-paced on line tutorials	14	10	4
Printed workbook	13	4	9
Lecture series (eg 1 hour per week for 10 weeks)	7	2	5
Special Interest group	5	4	1

What would encourage your participation in any training courses?

This was a free text question. Forty one responses were received and these have been groups according to theme. Some answers may have had more than one theme.

Theme	Responses
Time: Time to attend (4), Study leave (3), Rostered time/backfill of normal duties (11)	18
Support: Organisational/management (6), Financial (2)	8
Courses held in normal working hours	5
Official recognition via CME, professional development credit etc	5
Flexibility of times and locations	5
Ability to use skills immediately after course	5
Career path within pharmacy to enable use of newly acquired skills	5
Sufficient notification of courses to enable planning to attend	2
Follow up support to aid in using new skills	1
Workplace incentives	1
Minimal travel	1
Easy parking	1
Pay rise	1
Food	1

NEEDS ANALYSIS: Survey of staff enrolling in an Evidence Dissemination Service

Q1. How did you hear about the Evidence Dissemination Service	
I saw it in the CEs newsletter	8
I saw it advertised in the front intranet page	18
I saw a poster in the hospital	4
I was referred by a colleague	8
I work at another health service and a Southern Health employee referred me	0
Other	3
Missing Answers	5
Total	46
Other, please specify: direct email notification, was a part of the pilot phase, electronic newsletter, Received MMC email	

Q2. What is your role at Southern Health?	
Nursing	13
Allied Health	16
Medical	7
Other	10
Total	46
If Allied Health or Other, please specify: Physiotherapy 6, Occupational therapy 3, Strategic Planner/Manager SMICS 1, Pharmacy 2, Quality 1, Social work 1, Clinical psychologist 1, Speech pathology 1, Project Manager 2, Administrative 1, CCE 1	

Q3. In which Program do you work?	
Continuing Care	8
Corporate Office	1
Medicine Program	7
Mental Health Program	2
Support Services	2
Specialty Program	4
Strategy, Performance and Planning	1
Surgery Program	2
Women's and Children's	5
Other	13
Missing Answers	1
Total	46
If Support Services or Other, please specify: Nursing & Midwifery Education & Strategy, Research/Theatre, SMICS, Critical Care, Imaging guided therapy, Care in Context - HARP Program, SACS, General medicine, Capital Projects, Pharmacy, Anaesthesia, Ambulatory and Community Care, CCE	

Q4. At which Southern Health sites do you work?	
Kingston	5
Moorabbin	6
Clayton	24
Dandenong	8
Casey	5
Cranbourne Integrated Care	2
Other	5
Total	46
Other, please specify: All sites, Pakenham, Yarraman, Middle South CCU, Berwick	

Q5. Does your role involve decision making about introducing or changing use of TCPs?	
Yes	33
No	12
Missing Answers	1
Total	46

Q6. In your decision making around TCPs, approximately how often do you include evidence from research?

Never	0
Rarely	0
Sometimes	10
Often	10
Always	12
Missing Answers	14
Total	46

Q7. How often do you use the following resources to find information about technologies?

	Never	Rarely	Sometimes	Often	Always	Total
Personal subscription to journals	6	1	9	10	4	30
Personal Subscriptions to email list services	9	4	8	3	3	27
Library hard copy journals	3	7	15	2	1	28
The Cochrane Library	2	2	13	8	3	28
Other electronic databases of research	0	5	5	10	9	29
Guideline websites	1	8	12	4	6	31
Internet	0	1	12	11	7	31
Other	3	0	4	1	1	9
Missing Answers Total						14

Other, please specify: senior clinical staff, trade displays / meetings, conferences, in-service, other hospital guidelines, conferences

Q8. During the last 6 months, what is the average time you spent including information from research in your decision-making. Please indicate how long, on average, you spent searching for, accessing and appraising this information?

	<30 minutes	30-60 minutes	60-90 minutes	90-120 minutes	>120 minutes	Total
Searching	4	6	6	1	15	32
Accessing	5	6	5	2	12	30
Appraising	3	6	8	1	12	30
Missing Answers						14

Q9. Please rate your agreement with the following statements about evidence-based practice (EBP).

	Strongly Disagree	Disagree	Agree	Strongly Agree	Don't know	Total
Southern Health promotes the use of EBP	0	4	15	20	2	41
I believe EBP takes too much time	5	20	8	5	3	41
I know where to get Southern Health data for my decisions	3	14	19	4	1	41
I believe new medical technology does not require rigorous evidence to be introduced into clinical practice	19	18	1	4	0	42
I have access to research findings in my workplace	2	7	24	8	0	41
I believe EBP results in the best clinical care for patients	0	1	17	20	2	40
Southern Health facilitates employee's use of evidence in decision making for TCP change	1	8	24	3	4	40
I believe EBP is difficult	3	22	12	2	3	42
I believe that in the absence of research evidence EBP can still be applied to decision making about TCPs	3	14	14	3	7	41
Missing Answers						4

Q10. Please indicate how frequently you do the following

	Never	Rarely	Sometimes	Often	Always	Total
I consult a range of information sources	4	0	10	18	8	40
I include the views of consumers in my decision making	5	7	9	16	4	41
I use EBP guidelines or systematic reviews to change clinical practice where I work	4	1	15	20	2	42
I evaluate outcomes of practice change	2	7	13	15	4	41
I use evidence (research, clinical expertise, consumer preference) to change my clinical practice	2	0	15	18	7	42
Missing Answers						4

NEEDS ANALYSIS: Interviews with program, department and unit heads

The aim of this exercise was to pilot questions specifically for the Capacity Building Service with a small sample of decision-makers; 8 staff members were interviewed, 3 nursing staff, 4 medical and 1 support staff member.

What are the challenges to implementation?

Reach

- Dissemination to floor staff and night shift
- Limited access to computers for floor (nursing) staff

Buy-in

- Must involve everyone/ encourage broad buy-in. This will mean going to ward meetings etc
- Need to align values/aims b/w different parts of the hospital. Eg, hospital administrators care about finance, risk management and clinicians care about patients.
- Engage clinical champions

Resources

- Fit into existing processes and operational capacity
- Requires separate portfolio to implement each area
- Extra resources for training and credentialing
- Lack of program budget to implement
- Staff are already overworked: time to implement would be an issue
- Resources and support for training

Authority/ accountability

- Not a decision for NUMs
- There are issues about who is able to make decisions about technologies in other areas when they have no clinical knowledge **
- Will need to tailor framework to different levels of decision making eg. legislative requirements are the responsibility of the Quality Manager

Staff resistance

Paperwork/documentation

- Doesn't want to see excessive paperwork – must be user friendly and quick and easy to refer to eg. flowcharts
- Long application forms are a no-go. Generic forms get peoples' backs up so need to be tailored to specific cases so people can see the benefit of it

Communication Approach

- Need to focus on the way the message is sold and that people are made to feel part of the solution. Mandating a framework won't work. Need to present data re existing issues, and talk about how and why they have come about. This is then moved toward a potential solution
- Don't use jargon – don't talk like management

Scope

- Should be limited to certain, larger decisions

What support would be needed for implementation?

Tailored support

- Would be ward/department-specific
- Generic forms get peoples' backs up so need to be tailored to specific cases so people can see the benefit of it
- Posters
- Flowcharts (simpler than lengthy documents)

Training and Communication

- Computer skills (depending on the area)
- Must include really good communication
- Education/training as to why and how folk should use it. Also explain the research behind it and the benefits to both the individual and the organisation as a whole.
- Practical training will be needed to show people how to access data
- With regards to data - Must be sure to define the importance and relevance to people about what to do and why in order to get buy in

Scope (Integrated approach)

- Whole-of-unit focus so as to maximise skill sets and capacity
- Can't expect just one person to implement

- Organisation-wide focus for buy-in

Resources

- Need to create new positions to implement a framework
- Make ANUM and Junior registrar responsible for this – ie take some of their EFT. Learning about 'core business' (clinical risk) should happen earlier rather than later
- Additional assistance may be required from services such as CCE, the corporate office, ethics, and the strategy office
- Would require financial resources / backfill
- Separate, paid, non-clinical time (easier for NUMs)
- Would need additional EFT for project management as programs and units don't have spare people.
- [Some interviewees would prefer funds are spent on units to help them improve internal processes for clinical practice or research]

Documentation

- People will want templates or definitive documents with hints for using/completing. Documents must be succinct and use simple, plain language

What support or training is needed for decision making?

Three people said they didn't need any.

- One has an operations manager who can do it
- Already aware of how these processes work, and try to do it

Areas for support include

- Evidence (*n = 4*)
- Operational capacity
- Training and credentialing
- Financial implications (*n = 3*)
- Access and equity
- Legislative requirements (emphatic)
- Ethics
- Risk analysis (*n = 2*)
- Conflict of interest
- Fit with strategic plan (*n = 2*)
- Analysing and using data (*n = 5*)
- Culture change/ change management*

Will depend on the unit, profession and topic

- Med staff have more knowledge in reviewing evidence
- Risk analysis could be assisted by quality manager

What form of support is preferred?

Online training (*n = 5*)

- Electronic forms with 'hints'
- Self-paced resources are better for time poor clinicians
- Courses and resources
- One person specifically said they didn't want online
- Application guides on intranet which include links. Would include the 'nuts and bolts' of how to make applications at SH

Short course / workshops (*n = 3*)

- Can be easier to block out chunks of time
- At all sites
- Group learning has worked well in the past
- Good to work at the coalface
- Verbal discussion in group context can be good as it acts as peer support

Buy-in

- Need to get the main players to attend any workshops etc
- Need to tell some people (key decision-makers) that it is mandatory. If they think this is optional they won't do it.

"Always easier to get nurses to do training than doctors"

Discussion

Support for decision-making was needed in the areas of 'evidence' and 'analysing and using data'. Other areas requiring support were rated (from highest to lowest) as

- Financial implications
- Fit with Strategic Plan
- Risk analysis
- Legislative requirements

Three of eight respondents indicated they did not want or need support, while those who indicated a need for support stressed the need for it to be tailored to units' and professional needs.

Support for implementation primarily concentrated on education and training initiatives and increased resources. Computer skills and using data were two key examples of training needs while most suggestions for increased resources focused on EFT. Some comments regarding resource use include

- Making Assistant Nurse Unit Managers and Junior registrars responsible for implementation. "Learning about 'core business' (clinical risk) should happen earlier rather than later".
- Additional assistance may be required from services such as CCE, the corporate office, ethics, and the strategy office.
- "Separate, paid, non-clinical time" should be set aside. This was considered easier for Nurse Unit Managers to organise than medical staff.

Respondents also stressed the importance of tailoring support, and ensuring that any new documentation is specific, easy to use and quick to complete. Interviewees also commented that implementation should take place across the organisation and an integrated approach to communication efforts should focus on creating buy-in.

Most respondents (five of eight) indicated their preference for online resources as their preferred form of support. This included online forms with hints, application guides with links to other resources and include the "nuts and bolts" of how to make applications at Southern Health.

Three of eight interviewees preferred short courses and workshops as a methods for support, as some thought it easier to block out chunks of time for learning and sharing. One interviewee observed that "group learning had worked well in the past" with another commenting that "verbal discussion in group contexts can be good as it acts as peer support".

All interviewees commented that to get any form of support, effort was needed to create buy-in.

NEEDS ANALYSIS: Differences between professional groups

In their systematic review of information needs and information-seeking behaviour, Clarke and colleagues (2013) note the need for further investigation of the differences between health professional groups.[1] There were notable differences between medical, nursing, allied health and management/support groups in our study.

The interviews identified that medical and nursing staff make different types of decisions; nurses made more decisions about changing policies and protocols and fewer decisions regarding large equipment purchases and doctors reported the reverse. This is also reported by others.[2] Research evidence and local data were valued in decision-making for both groups; however nursing staff reported the use of local data more often than medical staff and medical staff noted the use of research evidence in guiding decisions more often than nurses. Further details are reported elsewhere.[3]

The surveys found that medical staff used systematic reviews and original research and accessed health databases and the Cochrane Library more often than nurses, and had higher levels of confidence for all aspects of finding, appraising and using evidence in decisions. Allied health staff came somewhere between the two for most findings. Further details are in the needs analysis findings above. These findings are consistent with others [1,4,5] but also in contrast to the review by Younger (2010) who found no significant differences.[6]

When selecting a preferred format for education in searching for, accessing and appraising evidence medical staff preferred self-paced online tutorials, nurses interactive workshops (eg ½ to 1 day), allied health staff short courses (eg 2-3 days) and the management/support staff had equal preference for lecture series (eg 1 hour per week for 10 weeks) and interactive workshops. The preferred formats for education in implementation of change were slightly different with medical staff still choosing self-paced online tutorials but nursing, allied health and other staff all preferring interactive workshops.

---

[1] Clarke MA, Belden JL, Koopman RJ, Steege LM, Moore JL, Canfield SM et al. Information needs and information-seeking behaviour analysis of primary care physicians and nurses: a literature review. Health information and libraries journal. 2013;30(3):178-90. doi:10.1111/hir.12036.

[2] O'Leary D F, Mhaolrunaigh SN. Information-seeking behaviour of nurses: where is information sought and what processes are followed? Journal of advanced nursing. 2012;68(2):379-90. doi:10.1111/j.1365-2648.2011.05750.x.

[3] Harris C, Allen K, Waller C, Hille V. Sustainability in Health care by Allocating Resources Effectively (SHARE) 3: examining how resource allocation decisions are made in a local healthcare setting BMC health services research. 2015;(Details TBA).

[4] Solomons NM, Spross JA. Evidence-based practice barriers and facilitators from a continuous quality improvement perspective: an integrative review. Journal of nursing management. 2011;19(1):109-20. doi:10.1111/j.1365-2834.2010.01144.x.

[5] Hider PN, Griffin G, Walker M, Coughlan E. The information-seeking behavior of clinical staff in a large health care organization. Journal of the Medical Library Association : JMLA. 2009;97(1):47-50. doi:10.3163/1536-5050.97.1.009.

[6] Younger P. Internet-based information-seeking behaviour amongst doctors and nurses: a short review of the literature. Health information and libraries journal. 2010;27(1):2-10. doi:10.1111/j.1471-1842.2010.00883.x.

DATA SERVICE: Interviews with staff responsible for collecting, maintaining and sharing data

Data sources

A total of 38 databases were identified by CIM; only those most relevant to organisational decision-making for resource allocation for TCPs were explored. A number of smaller databases for projects, audits or department-specific purposes were known to exist, however the extent to which data was stored and made accessible to others was unknown.

Representatives of 10 relevant departments/units were interviewed.

Table 1: Who has what data?

**Clinical Information Management (CIM)**<br>▪ Hospital activity data (separations) and reports<br>▪ Inpatient, outpatient, Emergency Department, theatre<br>▪ Waiting lists for elective surgery and mental health<br>▪ Some costing data	**Pathology**<br>▪ Inpatient and outpatient data<br>▪ Demographic data<br>▪ Episodic testing and results<br>▪ Billing information reported to Medicare
**Health Information Systems (HIS)**<br>▪ Patient record data eg demographic data, ICD 10AM codes, diagnosis (DRGs), medical history<br>▪ Waiting lists for inpatient admissions and elective surgery<br>▪ Emergency Department admissions<br>▪ Freedom of Information and Third Party	**Research Directorate**<br>▪ Research project and audit information, includes legal, financial, ethics etc<br>▪ Serious Adverse Events
**Clinical Risk, Quality Unit**<br>▪ Riskman data<br>− patient incidents<br>− staff near misses and incidents<br>− visitor near misses and incidents<br>− equipment<br>− complaints<br>▪ Medical Emergency Team (MET) and Code Blue (resuscitation) calls<br>▪ Coroner's information<br>▪ Clinical Review Panels	**Infection Control**<br>▪ Hospital-acquired infections eg MRSA/VRE<br>▪ Surgical site infections for high risk patients (cardiac, caesarean section, colorectal)<br>▪ Line infections in intensive care units<br>▪ Hand hygiene<br>▪ Flu vaccinations<br>▪ Staphylococcal bacteraemia<br>▪ Needle-stick injuries
**Clinical Audit, Quality Unit**<br>▪ Clinical audits on organisation-wide quality areas<br>− Medical record documentation<br>− Medication Safety<br>− Consent<br>▪ Hospital-wide indicators (from HIS data)	**Infectious Diseases**<br>▪ Notifiable diseases<br>▪ National antibiotics usage and surveillance program<br>▪ Cystic fibrosis database<br>▪ Microbiology data<br>▪ Antimicrobial resistance (AGAR)
**Diagnostic Imaging**<br>▪ Episodic patient data: Radiology information system (RIS) and Picture archiving and communications systems (PACS)<br>▪ Inpatient and outpatient data<br>▪ Three Cs (correct patient, correct procedure, correct site)<br>▪ Emergency Department procedures<br>▪ Source data<br>▪ Waiting times<br>▪ Interventional clinical data<br>▪ Public and private patient numbers<br>▪ Turnaround times<br>▪ Key performance indicators<br>▪ Number of incomplete procedures and not recorded data	**Pharmacy**<br>▪ Adverse Drug Reactions<br>▪ Patient discharge and dispensing data<br>▪ Do not have data for individual inpatients but do have<br>− S100 data (expensive, government funded)<br>− Expensive drugs<br>− Individual use eg lotions, inhalers, etc<br>− Some antibiotics<br>− Sterile products<br>▪ Special Access Scheme (for medications not approved by the Therapeutic Goods Administration for use in Australia)<br>▪ All drugs leaving pharmacy – legal requirement of the DPCS Acts and Regulations and the Schedule for the Uniform Scheduling of Drugs and Poisons (SUSDP)<br>▪ Non-PBS claims or 'off-label' items

Different types of data

- Aggregates and descriptive data of hospital activity (inpatient numbers, bed days etc)
- Patient Information
- Outcome data from projects and patients (eg SDR)
- Audit and primary research results relevant to Southern Health
- Qualitative data
- Performance information (eg financial)

Data collection and storage

Data can be collected routinely for all relevant patients or practices, or purposefully to address specific questions as they arise. Data can be collected by staff within the department/unit (primary) or be provided to the department/unit by others (secondary). Routinely-collected data in electronic systems can be transferred automatically between departments, other data such as survey responses, must be entered manually into locally developed databases.

Table 2: How is data collected and stored?

Department/Unit	Collection		Storage system
Clinical Information Management	Secondary	Routine	iPM/HOMER, Symphony
Health Information Systems	Secondary	Routine	iPM/HOMER
Clinical Audit, Quality Unit	Primary, Secondary	Routine	iPM/HOMER, Riskman, Birthing Outcomes System, Infection Control, local databases
Clinical Risk, Quality Unit	Primary, Secondary	Routine, purposeful	Riskman, plus local databases
Diagnostic Imaging	Primary, Secondary	Routine, purposeful	PACS-RIS, Riskman, Quality audits, local databases
Pathology	Secondary	Routine	2 x Laboratory Information Systems
Research Directorate	Primary, Secondary	Routine	DREAM, Merlin, HREC
Infection Control	Primary, Secondary	Routine	VICNISS, local databases
Infectious Diseases	Primary, Secondary	Routine, purposeful	Pathology Laboratory Information System, HOMER
Pharmacy	Primary, Secondary	Routine	Merlin, Riskman, Drug Usage Evaluations, local databases

Utilisation and decision making

Southern Health

Six units who collect data on their own activities used the data they collected for their internal, departmental decision-making. Common uses were benchmarking, service planning and equipment needs, practice improvement including quality and safety projects, financial and efficiency monitoring.

One unit noted that it operated on a strategic level providing information to the Chief Executive and the Board.

Health Information Systems data are mainly used by clinicians in patient care.

Eight units reported their data being used in other parts of the organisation. Many units collect, analyse and prepare reports for executive directors, program directors and department heads. People using data for decision-making on resource allocation include the Board, Chief Executive, Executive Management Team, Program Directors, Department Heads, Quality/Risk Managers, Finance Department Heads and Business Managers, Standing Committees and project steering groups, Quality Unit, Pharmacy and Infection Control department.

Requests

All units take requests for data with varying levels of demand for service. CIM responds to many requests, including most of those related to HIS. Others like the Clinical Audit unit have never had a request for information, but receive requests for help with audit tool design.

One interviewee suggested the information they collected would be useful to other departments, but thought others were unaware that they could make requests.

Pathology, Research Directorate, HIS and Infectious Diseases generally require ethics approval before providing data.

Requests to the Risk Manager are only granted for an organisation-wide decision and the request must come from a Director or Executive Director.

Some of the available data is accessible via the intranet and there is an expectation that decision-makers will access and interpret data for themselves.

Use in resource allocation decision making

Nine units gave examples of how data was used in decision-making for resource allocation of TCPs.

Table 3: Examples of data use in resource allocation decision-making

**Department/Unit**	**TCP Resource Allocation Use**
Clinical Information Management	Strategically and operationally eg organisational improvement and service enhancement<br>Service planning and equipment needs
Health Information Systems	Departments to make business case<br>Therapeutic Equivalence Program uses usage and patient data
Clinical Audit, Quality Unit	New projects or clinical practices<br>Make or review policies and protocols
Clinical Risk, Quality Unit	Capital planning eg. focus on falls – need to buy x number of low-low beds<br>Projects eg JMO safety checklist
Diagnostic Imaging	Used in evidence-based guidelines<br>One-off audits for research and decision-making, eg need for DR units (x-rays)
Pathology	Feedback trends in testing to hospital staff for them to review more thoroughly re. appropriateness of epidemiology
Research Directorate	Internal resource allocation by auditing number and type of research projects.
Infection Control	Units implement strategies or purchase new equipment to reduce infection rates.
Pharmacy	Projects eg Therapeutic Drug Monitoring, Traffic Lights, Therapeutics Equivalence, Medication safety

Reporting

Units regularly report data within Southern Health and external to the organisation. Some data collection and reporting to external bodies is mandated. Results are presented below.

Table 4: Internal and External Reporting of Data

Clinical Information Management (CIM)	Mandated	Pathology	Mandated
Managers (including CEO)	Yes	Pharmacy	Yes
External Registries eg Cerebral Palsy	No	State registries eg blood bank, donor register, transplant	No
Department of Human Services eg mortality data	Yes	Reference laboratories eg Microbiological Diagnostic Unit	No
		Peri-natal statistics	Yes
**Health Information Systems (HIS)**		**Research Directorate**	
WIES information and waiting lists to DHS	Yes	Therapeutics, HR	Yes
		Department of Human Services	Yes
		NHMRC	Yes
		Victorian Medical Insurance Agency eg Serious adverse events	Yes
**Clinical Risk, Quality Unit**		**Infection Control**	
Risk Register for Board/EMT	Yes	Chief Executive	Yes
Coroner’s office, Chief Psychiatrist, and Department of Human Services eg radiation safety and sentinel events	Yes	Program/Department Directors	Yes
		VICNISS (Department of Human Services)	Yes
		‘Vibes’ at the Austin Hospital for needle-stick injuries	No
**Clinical Audit, Quality Unit**		**Infectious Diseases**	
Joint Patient Quality and Safety Committee, Executive Management Team and Board Quality	Yes	Department of Human Services – notifiable diseases	Yes
Variance from hospital indicators to program managers	Yes	Australian Group against Antimicrobial Resistance	No
**Diagnostic Imaging**		**Pharmacy**	
Executive Director of Medical Services, Site MITs	Yes	Therapeutics Committee	Yes
Credentialing/Registration	Yes	Medication Safety Committee	Yes
		S100 drugs (Schedule 100 of the National Health Act)	Yes
		PBS (discharge/outpatient data for funding)	Yes
		Special Access Scheme	Yes

Other forms of dissemination

All but one unit had a means of distributing data internally. These were through:

- Data cubes on CIM intranet-site
- Quality indicators on the intranet
- Chief Executive’s Newsletter
- Quality and Risk Management Report (not public)
- Encapsulator (Pharmacy newsletter)
- Southern Health Research Report
- Quality of Care Report

Strengths of the current system

Reliable data – specifically for hospital indicators

- professional coding
- consistently collected and complete

Simple data collection tools eg. Survey Monkey

Reporting software (electronic)

- managers can self-serve
- includes templates and links to other processes, eg minutes and agendas for HREC
- easier, less time consuming
- DHS standardised tools eg. VICNESS, iPM/Homer

Staff skills in multidisciplinary teams for

- data collection (audit teams)
- data analysis and dissemination (CIM)

Easy access to data for use

- CIM website
- Scanned medical records
- Pathology LIS

Governance structure

- new reporting structure with COO
- used to report and share data eg representation of Pharmacy on multidisciplinary committees

Executive level support for reporting and using data

Collection of meaningful data able to be used in decision making eg Drug Usage Evaluations

Challenges for moving forward

Duplication of effort in data collection (due to not knowing what other people are doing)

No software or a lack of software

- inappropriate to storage, analysis and reporting needs
- slow and inefficient
- process for reporting is too manual
- (comment that HealthSmart has had limited success)

Unreliable and incomplete data eg financial

- gaps in data collection eg medico-legal
- definitions for measurement are inconsistent across the organisation eg EFT
- unrealistic expectations of reliability

Non-linked data limits access

- 'disjointed system'
- time consuming to find out where others' data is
- some database charge money for access eg Cardiobase
- would like access to BAR info to make business cases

Lack of timeliness for data availability

Lack of use of data in decision making and organisational planning

Limited governance of data systems

Difficulties getting staff buy-in for data collection

Lack of knowledge re use of data and expectations re what data can yield

Opportunities for improvement

Software improvement and investment

- central repository and linkages between systems
- automatic reporting
- increasing self-service options on web
- better software for housing and mining

Better governance needs

- oversight, ownership of data systems
- alignment of measures
- to agree on outcomes that reflect SH goals and capabilities
- 'business intelligence and good governance' to push through commitment to data usage

Education and Support

- how to use data – including trends overtime and effectiveness measures
- administration support for data software
- need more people within the organisation to be able to teach and support staff how to work with data

Data quality and quantity

- increase reliability and reduce gaps in data
- increase resources for EFT to collect data
- increase buy-in for collection and use in decision making

Issues generally related to factors contributing to the use of data in decision making. **Timeliness** and **ease of reporting** were facilitating factors for use. **Training and support** are needed for self-service options as they become more widely available. Need **to build awareness** of the need for, and existing opportunities of, data use in decision making for TCP resource allocation.

A large proportion of issues would be solved (or mediated) by **linking access to different data sets** and moving to **electronic systems** for housing, mining and reporting data.

While there is some overlap, users of an evaluation service will be different to those of a data service. Data needed in clinical decision making will differ to that needed for resources decision making and project work. These will also differ by operational or strategic needs.

CAPACITY BUILDING SERVICE: Evaluation Framework

The Capacity Building Service (CBS) is one of four services provided through the SHARE Program addressing healthcare decision-makers identified needs for using evidence and data in making and implementing and evaluating decisions on resource allocation for technologies and clinical practices (TCPs).

Aim of the service

To develop, implement and evaluate a service to train and support clinicians, managers and policy makers to use research evidence and health service data in decision-making and then apply it successfully in implementing and evaluating projects.

Activities

- Needs assessment
- Introductory session: What is evidence based practice? An introduction to the concepts
- Training workshops
  - Evidence-based change process
  - Evidence-based practice (EBP) (4 part course)
  - Introduction to implementation
  - Introduction to evaluation
  - Using evidence in decision-making
- Problem solving/support sessions
  - Finding evidence, appraising evidence and interpreting results
  - Project planning/implementation planning
  - Evaluation planning
- Online resources/teaching (to be developed)
  - Electronic workbook
  - PowerPoint presentation
  - Self-assessment quizzes

Pilot

Activities will be piloted with Pharmacy staff, members of medication-related committees and staff working on SHARE disinvestment pilot projects before wider implementation.

Purpose of the evaluation

The purpose of the evaluation is to provide information on the

- progress of the service and to offer areas for improvements
- effect of the service on knowledge, confidence and use of evidence in decision-making

Evaluation findings will be of interest to SHARE project managers to provide information on service quality and accountability. Findings will be added to the final analysis of the SHARE Program.

Stakeholders

Key stakeholders to be interested in the findings of the evaluation:

- Pharmacy staff
- Members of pharmacy-related committees
- People undertaking projects affecting resource allocation of TCPs
- Southern Health clinicians and TCP resource allocation decision-makers (including committees)
- SHARE Program Steering Committee
- CCE Evidence Service and Evaluation service
- Victorian Department of Human Services

Key evaluation questions

Key questions have been modelled around the RE-AIM framework for health program evaluation.

Reach

- How many people participated in person and online?
- What percentage of participants was from the target group?
- What percentage of participants attended more than one course?

Effectiveness

- To what extend has the service changed participant's (measures dependant on course attended)
  - Knowledge and skills
  - Confidence
  - Use of evidence and data
  - Implementation practice
  - Evaluation practice
- How are these changes affected by the amount and type of classes attended?
- How useful and appropriate were the sessions/materials to current practice?
- What best helps participants with confidence/practice change?
- What was most useful in the follow-up session?

Adoption

- Has there been a demand for the service outside the target group?
- What is the potential for spread to other health services?
- Has there been any adaptation of the resources by participants?

Implementation

- To what extent was the service delivered as planned?
- To what extent were participants satisfied with the service and the materials supplied?
- Are there areas of EBP participants feel are missing from the service?

Maintenance

- How have measures of behaviour change (use), knowledge and confidence been sustained over time?
- What is the potential for service sustainability?
- What are the costs associated with delivery of this service?

Assembly of evidence

Data will be collected from participants using a before-after-after design. Quantitative and qualitative information will be collected through the use of surveys administered at the time of service delivery and via email for follow-up.

Changes in EBP knowledge and skills will be assessed with the previously validated Fresno test.

Information on spread and sustainability will be collected by utilising questions used previously in other SHARE evaluation activities.

Budget and resources

Resources and funding come from the SHARE Program.

SHARE project staff will design, implement and evaluate the service, with the exception of the evaluation training and support sessions which will be designed and delivered by the CCE Evaluation Consultant.

Limitations

- Only three months follow-up in pilot
- Self-report bias for frequency and confidence. Using questions that might make people feel judged and over report use, frequency or confidence
- Trialling adapted Fresno test

Ethical consideration

All evaluation activities as part of SHARE have received prior ethics approval from Southern Health as quality assurance activities.

Dissemination and utilisation

A report outlining the findings will be distributed to

- SHARE Program Steering Committee
- Southern Health EMT

Findings will be incorporated into the final SHARE Program report which will have a wider dissemination.

Evaluation Plan

Domain	Key evaluation questions	Success Indicators	Source of data	Method of collection	Timing of collection
**Reach**	How many people participated (online and in person)?	N/A	Attendance records	Document audit	Each workshop
	What percentage of participants were from target group?	N/A	Attendance records	Document audit	Each workshop
	What percentage of participants attended more than one class?	>65%	Attendance records	Document audit	Each workshop
**Effectiveness**	To what extend has the service changed participant's<br>▪ knowledge and skills in finding and appraising evidence	Increase in test scores (>80%)	Workshop participants	Fresno test	Before/after/3 mths after
	▪ knowledge and skills in using evidence, implementation and evaluation	Increased knowledge & skills	Workshop participants	Questionnaire	Before/after/3 mths after
	▪ confidence	Increased confidence	Workshop participants	Questionnaire	Before/after/3 mths after
	▪ use of evidence and data in decision-making	Increased frequency	Workshop participants	Questionnaire	After/3 months after
	▪ implementation practices	Increased frequency	Workshop participants	Questionnaire	After/3 months after
	▪ evaluation practices	Increased frequency	Workshop participants	Questionnaire	After/3 months after
	How are these changes affected by the amount/type of classes attended?<br>How have online materials supported learning?	N/A	Workshop participants	Questionnaire	After/3 months after
**Adoption**	Has there been a demand for the service outside the target group?	Inquiries, requests for courses	CCE Admin/Records	Document audit	End of project
	What is the potential for spread to other health services?	N/A			
	To what extent has adaptation of the resources or service occurred?	Adaptation	Key participants	Questionnaire	End of project
**Implementation**	To what extent was the service delivered as planned?	Fidelity	Implementation plan	Audit	End of project
	To what extent were participants satisfied with the service and materials?	>80%	Workshop participants	Questionnaire	After workshops
	Are there areas of EBP participants feel are missing from the service?	N/A	Workshop participants	Questionnaire	3 months after
**Maintenance**	Over what time period are changes in knowledge, confidence and behaviour change sustained?	Sustained change	Workshop participants	Questionnaire	3 months after
	What is potential for service sustainability?	Estimate of resources requires for replication<br>Additional funding	Resource audit<br>Project contract/budget	Audit<br>Audit	End of project

Timelines

	Year 1												Year 2																					
	July		Aug		Sept		Oct		Nov		Dec		Jan		Feb		Mar		Apr		May		Jun		July		Aug		Sept		Oct		Nov	
Evaluation framework and plan draft	■	■																																
Evidence based change process		■															■																	
What is evidence based practice?				■														■																
Evidence based practice – Part 1					■														■															
Evidence based practice – Part 2					■														■															
Evidence based practice – Part 3						■														■														
Evidence based practice – Part 4						■														■														
Using Evidence in Decision Making						■	■													■	■													
Introduction to implementation							■														■													
Introduction to evaluation								■					Follow-up									■							Follow-up					
Costing data collection																		■	■	■	■	■	■	■	■	■	■	■	■	■				
Support Session: Evidence							■		■		■		■							■		■		■		■								
Support Session: Planning/implementation									■		■		■							■		■		■		■								
Support Session: Evaluation								■		■		■		■							■		■		■		■							
Support Session: Guidelines and procedures								■		■		■		■							■		■		■		■							
Data analysis		■	■	■	■	■	■	■	■	■	■	■	■	■					■	■	■	■	■	■	■	■	■	■	■	■	■			
Pilot Report															■	■	■																	
Final Report																															■	■	■	

CAPACITY BUILDING SERVICE: Evaluation Results

Evaluation methods

CCE collected baseline data from all participants at the beginning of workshop. All participants were asked to complete a feedback form at the conclusion of each workshop, with the four-part EBP Workshop treated as one workshop.

In addition, the participants at the EBP Workshop completed a modified Fresno test for EBP knowledge at the beginning and end of the course. The Fresno test for EBP knowledge is validated for use in medical graduates and is a short-answer test. It estimated that each test takes more than twenty minutes to mark which is very resource intensive. A multiple choice version was developed in collaboration with a university-based colleague who teaches EBP to medical students. The multiple choice version was used to assess knowledge in our largely pharmacy-trained population with a view to developing a tool for use in future mixed population training courses.

Three months post workshops participants were also asked to complete a feedback form, and in the case of EBP workshop participants, also complete the modified Fresno test.

Quantitative and qualitative information was collected via questionnaires containing multi-choice answers, open ended questions, Likert scales and checklist responses.

Data limitations

Participants were asked to provide a unique confidential identifier made up from their day of birth and the last four digits of their phone number. The intention was to maintain confidentiality while allowing CCE staff to compare results. Although the baseline questionnaire had detailed instructions and a worked example of how to create the identifier, follow-up questionnaires had an abbreviated example. This abbreviated example may not have been sufficient as some participants created different identifiers on their follow up sheets. This meant that we were unable to match the before and after responses for all participants.

There were also slight variations in some of the evaluation questions across the range of workshops. This was due to involvement of several staff members in designing and editing surveys and identifies the need to define a consistent set of evaluation questions to be used in a suite of training programs.

Reach

Four workshops were delivered and seven follow-up sessions were offered. Baseline data was collected from participants at the first workshop they attended. Across the four workshops 77% (17/22) of participants provided baseline data.

Recruitment

Recruitment was targeted at staff working in the Pharmacy Department and members of medication-related committees. It was recognised that these staff regularly made decisions or were involved in projects regarding a change in use of a TCP. To promote the program, an introductory talk on EBP was held at a Pharmacy meeting; 37 participants attended.

In addition, staff involved in SHARE pilot disinvestment projects were invited to attend.

Participants

Twenty-two participants completed one or more of the courses. Half were from Pharmacy (11/22), four were nurses, one was an allied health professional and six did not specify their discipline. Participant's roles at Southern Health are represented in Table 1. Half of the 22 participants (11/22) attended more than one workshop.

Two participants engaged in the follow-up sessions. No data was collected to determine the usefulness of these sessions.

Table 1: Participant's role at Southern Health

	Pharmacy	Nursing	Allied Health	Unspecified	Total
Evidence-Based Change Process Workshop	5	1	1		7
Evidence-Based Practice	8	3			11
Introduction to Implementation	4	2		2	8
Introduction to Evaluation	5	1		3	9

The majority of participants were from the target population. Four participants reported that they were not involved in any of the activities associated with the introduction of TCPs at Southern Health, but they did indicate that they would in the future.

Table 2: Participant's role at Southern Health with relation to TCPs

	More than one answer allowed (N = 17)
I source and appraise evidence to support changes in practice	9
I am involved in making decisions regarding introduction of or change in use of TCPs	11
I implement practice change	13
I evaluate practice change	9
I don't currently do any of the above now but will in the future	4
I don't currently do any of the above now and won't in the future	0

Effectiveness

Sources of information

Participants were asked to rate how often they used the resources listed in Table 3.

Table 3: Use of information resources at baseline

	Never	Rarely	Sometimes	Often	Always	Often or Always	Total
Clinical practice guidelines			3	8	6	14	17
Colleagues		1	3	6	7	13	17
Southern Health data		5	6	4	2	6	17
Original research		3	6	6	2	8	17
Systematic reviews		6	5	4	2	6	17
Consumers	1	12	2	1		1	16
Other	1	2	6	4	1	5	14
Specify Other		1	3	3	1	4	8

Most participants used clinical practice guidelines and colleagues as their main sources of information. This is a similar finding to the survey of senior decision-makers at Southern Health. It is also interesting to note that the majority of participants rarely utilised consumer opinion to inform their practice.

Participants who answered 'other' were asked to specify the additional sources of information. Six of the 14 participants did not provide examples. The following examples were given by eight participants:

- Rarely: Hospitals and drug information centres
- Sometimes: Reference books and websites eg Micromedix and EviQ, conferences, other health services
- Often: Other health services, conferences, Department of Human Services
- Always: Conference presentations, contacts at other hospitals

At three months follow up participants were again asked where they sourced information for TCP projects. Responses are shown in Table 4. It is difficult to comment due to the low response rates.

Table 4: Use of information resources at 3-month follow-up

	Never	Rarely	Sometimes	Often	Always	Often or Always	Total
Clinical practice guidelines			2	4	1	5	7
Colleagues			1	2	4	6	7
Southern Health data	1	1	2	2	1	3	7
Original research		1	2	2	2	4	7
Systematic reviews		1	2	2	2	4	7
Consumers	3	3		1		1	7
Other	1		3	2	1	3	7
Specify Other					1	1	1

Workshop 1: Evidence-based change process

Seven participants included five from Pharmacy, one nurse and one allied health clinician. Four CCE staff also attended the workshop for training.

Understanding of EBP

Participants were asked how they define EBP and to specify if they had undertaken any previous training. This question was only asked at the EBP Change Process workshop. The following six definitions were given.

- "How we should be practicing clinically everyday"
- "Searching for and identifying evidence to support practical change"
- "Practice which incorporates literature/consensus, needs of the person/population and expertise of the clinician"
- "Using available evidence from the literature, clinical expertise and consumers to influence how things are done"
- "Using the best available evidence be it RCT, expert opinions or Cochrane"
- "Practice based on the best available information from sources including published literature, expert consensus, public and local data"

One participant had undertaken training in EBP "many years ago" but did not disclose the type of training and another participant "completed some aspects at Robert Gordon University, Aberdeen Independent Prescribing Course". The two staff members who correctly answered by referring to research, clinical expertise and patient/consumer perspectives had not had previous training

Evidence-based change process

The majority of participants felt that they were 'not very' to 'moderately confident' with the principles of an evidence-based change process model prior to the workshop, and several were 'not confident at all'.

Table 5: How confident are you at the following? (baseline)

	Not at all confident	Not very confident	Moderately confident	Quite confident	Very Confident	Total
Assessing the need for change		1	2	3		6
Developing a change proposal		2	3	1		6
Implementing change	1	5				6
Evaluating change	3	3				6
Involving consumers	1	5				6
Using action research processes		4	2			6
Undertaking literature reviews	1	1	3	1		6
Undertaking barriers and enablers assessment	1	3	2			6

Levels of confidence increased after the workshop and were maintained at the 3 month follow up.

Table 6: How confident are you at the following? (3 month follow up)

	Not at all confident	Not very confident	Moderately confident	Quite confident	Very Confident	Total
Assessing the need for change			3	3		6
Developing a change proposal		2	2	2		6
Implementing change			4	2		6
Evaluating change	1	2	1	2		6
Involving consumers		5	1			6
Using action research processes	2	2	1	1		6
Undertaking literature reviews		2	1	2	1	6
Undertaking barriers and enablers assessment			3	2	1	6

At the end of the workshop all participants reported that their knowledge had improved.

Workshop 2: Evidence-based practice

This workshop was delivered as 4 half-day sessions. Eleven participants attended; five from Pharmacy and 3 nurses.

Knowledge and skills

Knowledge and skills were assessed using the modified Fresno test. The maximum possible score is 27.

The average score for the 11 participants who completed the test before the workshop was 17.9 (66.3%), which increased to 18.3 (67.8%) after the workshop. The average score for the 5 participants who completed the test at 3 months was 20.2 (74.8%). Although this figure is higher than that of the before and after tests, it may be due to the fewer number of participants who completed it; the 5 respondents may have had higher scores in the earlier tests.

This trial of the adapted multiple choice Fresno test did not show an increase in the participants' skills after a short course in EBP skills. Baseline scores in the published literature are lower than our baseline scores of 66%, ranging from 17% to 54% of total possible score. Improvements after training are higher than our 1.5% improvement, ranging from 6.5% to 46%. This could be because the adapted test is no longer valid and reliable or perhaps SHARE participants had higher baseline knowledge of EBM concepts than those in other studies therefore the course was reinforcement or consolidation of concepts rather than new learning.

Confidence

Most participants did not feel very confident with regards to undertaking the tasks associated with finding and appraising research evidence. This trial of the adapted multiple choice Fresno test did not show an increase in participants' skills after a short course in EBP skills which conflicts with the participant's self-reported increase in confidence levels in all concepts of EBP.

Table 7: How confident are you at the following? (baseline)

	Not at all confident	Not very confident	Moderately confident	Quite confident	Very Confident	Total
Asking an answerable question		6	3	2		11
Searching databases (eg Cochrane and PubMed)	1	7	3			11
Accessing full text articles	1	3	4	2	1	11
Appraising the quality of an RCT	2	6	3			11
Appraising the quality of a systematic review	2	5	4			11
Identifying elements of bias	2	5	4			11
Interpreting common statistical values	5	6				11
Assessing principles of study design	3	5	3			11

The levels of confidence increased considerably at the end of the 4 session workshop but dropped back slightly for the 5 respondents who completed the 3 month review. The small numbers make it difficult to draw further conclusions. There is missing data for appraisal of RCTs.

Table 8: How confident are you at the following? (immediately after workshop)

	Not at all confident	Not very confident	Moderately confident	Quite confident	Very Confident	Total
Asking an answerable question			2	5	4	11
Searching databases (eg Cochrane and PubMed)			4	6	1	11
Accessing full text articles			4	3	4	11
Appraising the quality of an RCT		2	3	5		10
Appraising the quality of a systematic review		1	7	3		11
Identifying elements of bias			5	6		11
Interpreting common statistical values	1	1	7	2		11
Assessing principles of study design		1	5	5		

Table 9: How confident are you at the following? (3 month follow up)

	Not at all confident	Not very confident	Moderately confident	Quite confident	Very Confident	Total
Asking an answerable question			2	2	1	5
Searching databases (eg Cochrane and PubMed)		1	2	1	1	5
Accessing full text articles		1	1	2	1	5
Appraising the quality of an RCT						
Appraising the quality of a systematic review			4	1		5
Identifying elements of bias		1	3	1		5
Interpreting common statistical values		2	2	1		5
Assessing principles of study design		2	2	1		5

Four of the five respondents said they had used the skills learned from the workshop in the 3 month period and one expected to use these skills in the following 6-12 months.

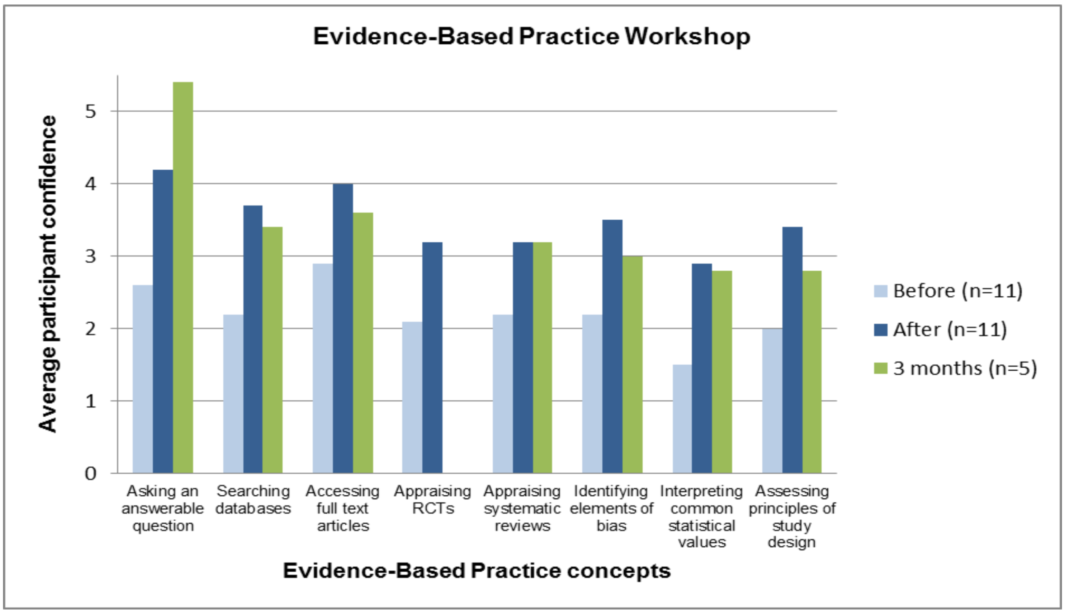

Workshop 3: Introduction to implementation

Eight participants attended; 4 from Pharmacy, 2 from nursing and 2 unspecified.

Participants were asked at the end of the workshop, and again at 3 month follow up, to rate their improvement in knowledge and confidence to undertake the activities of evidence-based implementation. This was graded from 1 = no difference to 5 = significant improvement.

All participants reported considerable improvements in both knowledge and confidence levels, and while improvements were maintained at the three month follow-up, these levels had somewhat reduced. This may have been due to a loss of knowledge and confidence or perhaps because only 5 of the 8 attendees responded at 3 months. These 5 participants may have had lower than average scores after the first workshop and maintained these levels at 3 months. All 5 reported that they had applied these skills in the three month period.

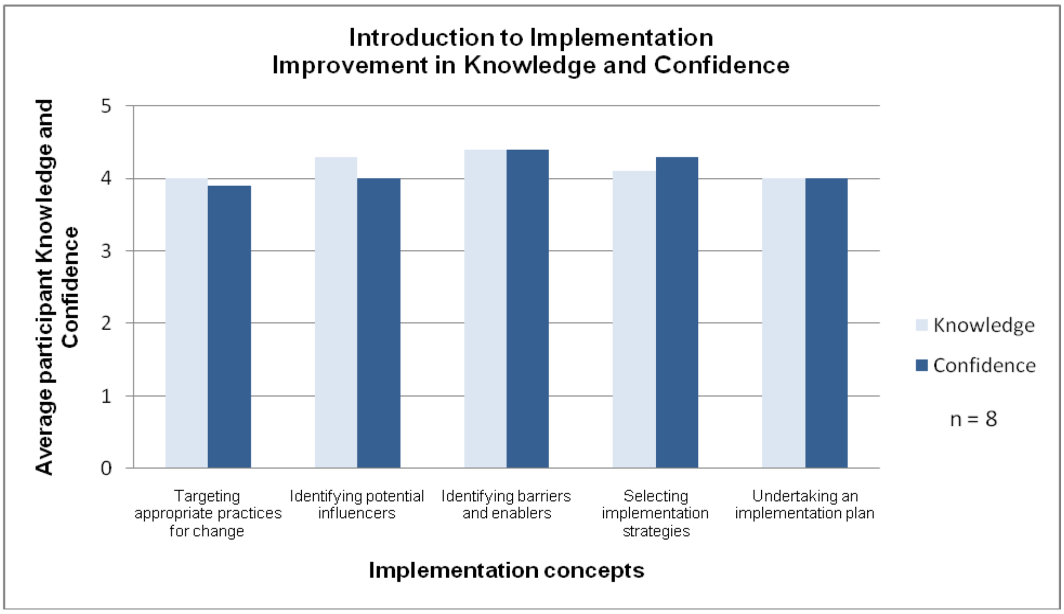

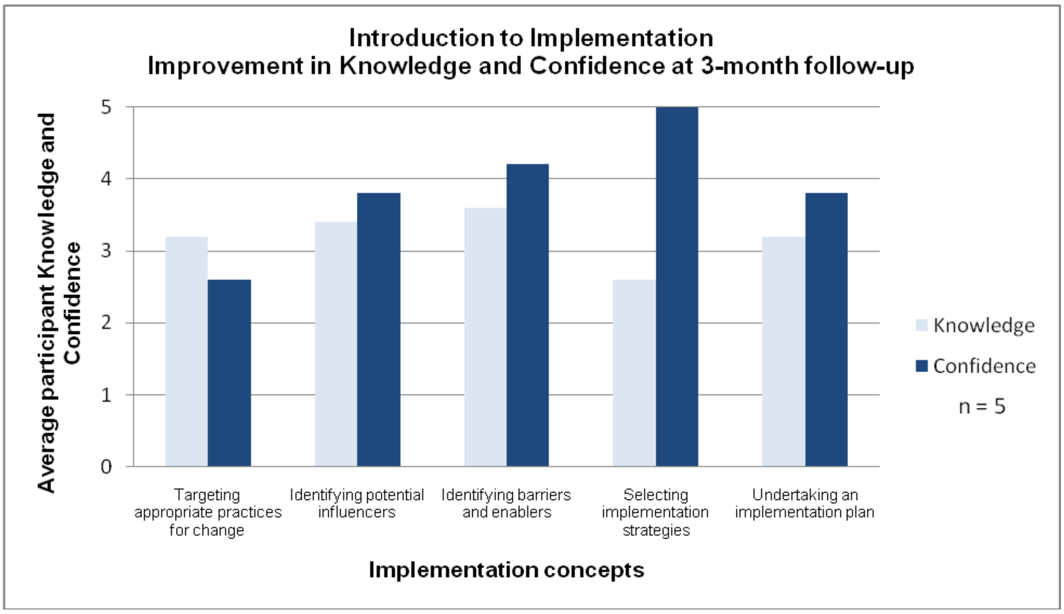

Workshop 4: Introduction to evaluation

A total of nine participants attended; 5 from Pharmacy, 1 from nursing and 3 unspecified.

All participants' knowledge and confidence improved after the workshop; however only 2 participants completed the 3 month follow-up. One respondent had used their skills in evaluation and the other commented that they planned to do so in 3-6 months.

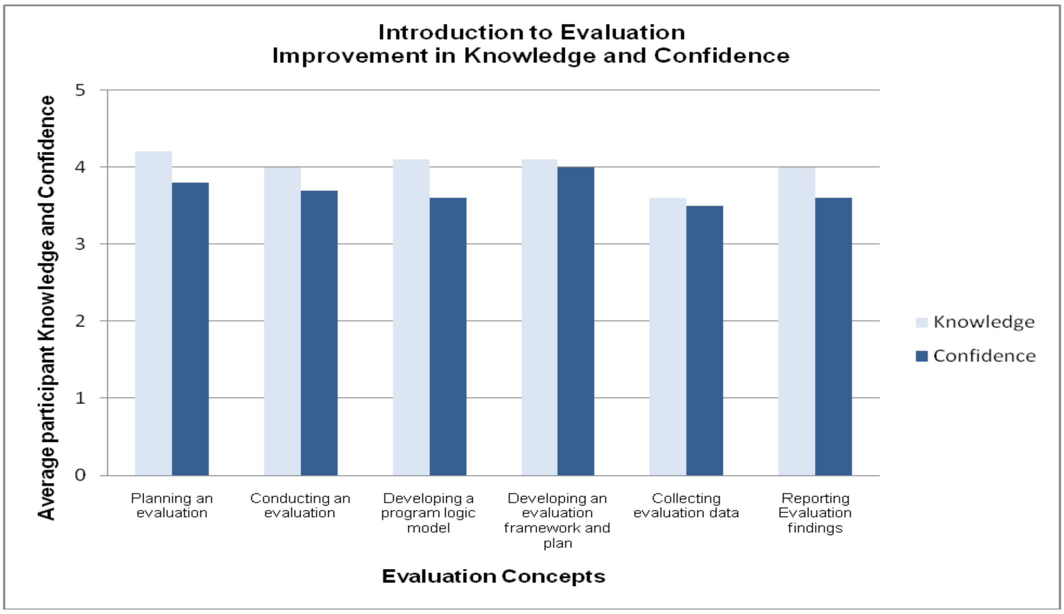

Problem solving/support sessions

To provide ongoing support to those who attended the workshops, an ongoing rotating four-weekly series of open workshops were offered. Participants could seek assistance from CCE staff in the relevant topic area, and share their learnings and develop networks with colleagues.

Week 1: Finding and appraising evidence and interpreting results

Week 2: Project planning and implementation

Week 3: Evaluation

Week 4: Guidelines and protocols

Only two participants attended the open workshops over the first two months. The program was discontinued and no evaluation was undertaken.

Adoption

No activity was undertaken beyond the pilot groups.

Two participants (members of organisation-wide committees) noted that the CBS should be available to all Southern Health staff.

Two pharmacists requested a copy of the 'What is evidence-based practice? An introduction to the concepts' presentation in order to make the slides available to staff members who could not attend.

A link to the CCE website resource page containing workbooks on how to find evidence was distributed to Pharmacists.

Implementation

Fidelity

The four workshops and support sessions were delivered as planned. Funding for the final year of SHARE Program was withdrawn. The workshop on 'Using Evidence in Decision-Making' and the associated online resources had not been developed at this time so were not delivered.

Participant satisfaction

Responses were very positive.

Table 10: Experience of the Evidence-Based Change Process Workshop (n = 7)

	Strongly agree	Agree	Neutral	Disagree	Strongly disagree
The session was relevant for my needs	1	6			
I found the subject matter and content interesting	2	5			
I learnt nothing of value				2	5
The education methods used were effective		7			
There were adequate opportunities for interaction and discussion	2	5			

Table 11: Expectations of the Implementation and Evaluation Workshops (n = 8 and 9)

	1<br>Did not meet my expectations	2	3	4	5<br>Exceeded my expectations
Implementation			1	5	2
Evaluation				8	1

Participant feedback

Participants were asked to comment on what they liked most about the session, what they liked least (Evidence-Based Change Process Workshop only), suggestions for improvement and suggestions for follow-up topics or other courses (Table 12). Across the four workshops, common themes were revealed.

Overall, participants appreciated the interactive and hands-on format of the sessions. They felt that the formats were clear and straightforward and welcomed the opportunities for discussion. Participants also liked the examples presented by CCE staff and the insight they provided from learnings in other projects.

When asked what they liked least about the Evidence-Based Practice Change Process Workshop, two participants highlighted that the focus on *"highly-resourced projects (although excellent) are not very applicable to small projects"*.

Participants suggested that more time to discuss their own projects and to apply the theory they had learned in the sessions to these projects would have been beneficial across the four workshops. Other suggestions for improvement included focusing presentations on smaller projects where provisions of resources are limited. It was also suggested that presenters identify two projects to workshop per session.

Participants thought that the Evidence-Based Practice Workshop could be improved by including a session on how to search using Medline. They also felt that providing pre-workshop reading materials would have aided their learning.

Participants who attended the Introduction to Evaluation Workshop suggested that provision of an evaluation report template would have been useful. They also suggested that an additional session where they would be able to apply what they had learned in a current project would be helpful.

Project management, barrier and enablers analysis, undertaking literature reviews, journal clubs, statistics and program logic mapping were suggested by participants as future activities.

Maintenance

Costing was planned but not completed due to loss of funding in the evaluation phase of the SHARE Program.

Table 12: Participant Feedback

	Evidence-Based Change Process Workshop	Evidence-Based Practice Workshop	Introduction to Implementation Workshop	Introduction to Evaluation Workshop
**Liked most**	▪ Small group and discussion opportunities<br>▪ Discussion about individual projects, tips from previous learning's<br>▪ The worked examples presented<br>▪ Applying the content of the sessions to an actual project<br>▪ Interaction<br>▪ Gave a good framework for change process - formalised how to go about the change process<br>▪ Relevant content and straight forward format	▪ Able to listen without copying down notes, nice and casual environment, hands-on<br>▪ Interactive nature, handson search<br>▪ Nice small group, interactive and hands on<br>▪ Very friendly environment, find it easy to ask questions<br>▪ Last session very worthwhile, second session with computers very good<br>▪ Informative and practical, very useful in doing research<br>▪ Good order of topics, lots of hands on (lit searching, minties/coins, article appraisal), didn't finish late, and clear presenting by Kelly<br>▪ The online tests were good, got me thinking, revision of previous study I have done ie refresh, plus new knowledge<br>▪ How to assess articles<br>▪ The introduction on how to start formulating a clinical question, time for questions<br>▪ Way things were explained	▪ Good theoretical background and practical examples<br>▪ Very informative and applicable to my practice<br>▪ Given a handout at the start<br>▪ Workshopping sections helped to demonstrate the points<br>▪ It is practical and applicable, easy to follow<br>▪ All brilliant, the most relevant and useful for me so far<br>▪ Examples, interactions	▪ Presenters enthusiasm<br>▪ Very interactive and informative<br>▪ Explanation of program logic<br>▪ Aimed at the level of knowledge of attendees. Very easy to follow<br>▪ Program logic. Opportunity offered for future support<br>▪ Continuity, sequential<br>▪ Use of medication strategy as an example<br>▪ Learning about how we should be doing evaluation compared to what we actually do in practice<br>▪ Presented at an introductory basic level. Inclusion of samples and templates. Provision of slides
**Liked least**	▪ Large emphasis on highly-resources projects (although excellent) not very applicable to small projects<br>▪ No food at morning tea<br>▪ Resource issues is a problem in being able to continue to give good support tools for the projects and future projects	▪ Not Asked	▪ Not Asked	▪ Not Asked
**Suggestions for improvement or any other comments**	▪ Provide examples from smaller projects<br>▪ List of other related sessions that are available with a suggested order for attending them so that you get the most out of each session<br>▪ Allow time to discuss steps 2, 3, 4	▪ Looking at more articles and practical application of knowledge<br>▪ Handouts at the start of the session - ask us not to look at answers until discussing. Then can write additional information on handout	▪ Identify two projects to workshop	▪ discuss how to put together an evaluation report<br>▪ Too short to learn and feel confident about how to do an evaluation but probably something you need to actually do to learn it.

	relevant to own projects, include more information about support and additional information services available through CCE	▪ I believe people will gain more if they have access to the next session slides or materials so that they can read in advance<br>▪ Combine first and second sessions into 1 afternoon, give out handouts before session starts, focus more on appraising and statistics, a lot of the statistics and terms were not covered in the tutorials<br>▪ Maybe include 'Medline' in this course would be useful<br>▪ Combine first two sessions into one and spend third session (before appraisal session) on reading the article - very difficult to plough through the terms, stats, significance statements. Still don't quite feel confident in understanding statements made by authors/whether results are statistically significant etc<br>▪ Some of it was rushed unnecessarily eg the more difficult numbers stuff confidence intervals<br>▪ A bit longer on assessing articles<br>▪ Maybe a bit of pre-reading about types of studies and a bit of background about the same statistics (P, Abs Risk, CI).<br>▪ Handout notes without answers - so we can write our own answers on		▪ If larger workshop time, apply the information to procedures that attendees are currently working on.
**Suggestions for follow-up topics or other courses relevant to professional development**	▪ The easy to follow guide for clinicians for EBP<br>▪ Basic concepts of project management<br>▪ Barrier and enabler analysis, change implementation<br>▪ How to do literature searches<br>▪ Implementing change, evaluating change	▪ Would really like sessions where Kelly could read bits through sections of an article bit by bit<br>▪ More of the same sort of thing. Access to journal club with an expert to lead the club, when we are experienced we could lead our own clubs in our clinical areas<br>▪ More on P values and Confidence Intervals<br>▪ Application of what we learned	▪ Evaluation<br>▪ Project management in general<br>▪ Lots more of similar, support for projects and submissions<br>▪ Follow-ups	▪ Bring our own evaluation with us to improve and determine type of evaluation or perhaps better ways to ask questions for evaluation.<br>▪ Project management<br>▪ Please put on a program logic study day that we can attend.

PROJECT SUPPORT SERVICE: Factors influencing the SHARE pilot disinvestment project

WHAT WORKED?	WHAT/WHO HELPED?	WHAT DIDN'T WORK?	HOW COULD IT BE IMPROVED?
**Application process**			
▪ Working on application with another person<br>▪ Diary (keeping note of time used on project application	▪ CCE Director<br>▪ Medical Program Director<br>▪ Finance Director<br>▪ Business manager<br>▪ Other clinical staff<br>▪ Manufacturer's information	▪ Application process too lengthy<br>▪ Time factor (slow/hard to coordinate when working with other hospitals)<br>▪ Department of Human Services (DHS) changed the rules<br>▪ Difficulty in understanding memos from DHS	▪ Being aware of the work load prior to commencing work<br>▪ Having more time dedicated towards the application process as well as planning and implementation of changes (lead staff are involved in the application process while other clinical staff voluntarily take up the extra clinical load. Backfill for clinicians involved in projects)
**Implementation**			
▪ Small workshops with medical teams<br>▪ Change of DHS staff (more clarity in information)	▪ CCE Project Officer<br>▪ Medical Directors (Surgery Program, General Medicine, Plastics)	▪ Having to repeat training every 3-6 months due to staff rotations<br>▪ Involvement of other hospitals with staff who are not dedicated/committed (eg. disputes amongst doctors from another site)	▪ Having full funding (Not receiving full funding leads to belief that applicant should over-inflate costs. Or DHS should do their own costing)<br>▪ Should have performed barriers and enablers analysis earlier
**Ongoing service**			
▪ Maintenance of a booking system<br>▪ Quarterly meetings with all services (hospitals)		▪ As above	▪ Having a single dedicated treatment room (No dedicated treatment area increases the time for preparation and cleaning. Clinical time is small in comparison to set up/clean up time)<br>▪ Adequate ventilation (clinicians feel ventilation in rooms may be inadequate as aerosols are created with treatments)
**Reporting**			
▪ CCE assistance with Access database for reporting and help to coordinate reports between TCPC and DHS	▪ CCE Project Officer<br>▪ Having a person in charge of data entry	▪ "Shifting the goal posts" by DHS re what reporting they want	▪ Clearer and more comprehensive DHS templates
**Other**			
▪ Though staff leave and secondments are difficult there can also be an advantage of working with other staff who become familiar with the project		▪ Accountability of other sites<br>▪ "Buy-in" from other hospitals throughout the entire process<br>▪ Staff secondments and/or leave (can be hard to enter part way through and person you are replacing is not always contactable)<br>▪ SH systems: Procurement delivered machine on Friday afternoon so it was left in the corridor over the weekend. Electronic (bio-testing) cord was lost and a new cord had to be purchased<br>▪ Joint application (this project had many initial partners). Would only do a joint application in future with 1-2 other hospitals<br>▪ As the lead applicant, SH should have been consulted on the choice of sites funded ie. 8 hospitals applied and 4 were funded	

FACTORS THAT INFLUENCED DECISION-MAKING FOR SUPPORT SERVICES

	EVIDENCE SERVICE			DATA SERVICE			CAPACITY BUILDING SERVICE			PROJECT SUPPORT SERVICE		
	Identify, capture and process synthesised evidence	Translate into user friendly formats	Disseminate to decision-makers	Identify high risks and variations in practice	Translate into user friendly formats	Disseminate to decision-makers	Provide training in accessing and using evidence and data	Provide training in implementation & evaluation	Mentor and support	Provide advice regarding methodologies and methods	Assist with project development & administration	Assist with data capture, data entry and analysis
**BARRIERS**												
Lack of time and opportunity [7-20]			✓			✓					✓	✓
Lack of skills [7, 9, 11-15, 17, 19-26]	✓	✓	✓	✓	✓	✓	✓	✓	✓	✓	✓	✓
Lack of confidence [12, 27]	✓	✓	✓	✓	✓	✓	✓	✓	✓	✓	✓	✓
Lack of interest or competing priorities [14, 20, 27-29]			✓			✓	✓		✓			
Lack of awareness of research and data [7, 12, 17, 21, 25, 28]	✓		✓	✓		✓	✓					
Lack of use of available research and data [21, 24, 25, 28, 30]		✓	✓		✓	✓	✓	✓	✓	✓	✓	
Lack of relevant research and data [9, 10, 14-16, 18, 19, 21, 22, 31] particularly for disinvestment [19, 20, 23, 32, 33]	✓			✓			✓					
Poor quality of health data [10, 19, 21, 24, 31, 34, 35]				✓	✓	✓						
Unfamiliar or difficult to use formats of research and data [12, 17, 19-21, 24, 31]		✓			✓		✓					
Lack of policies and interventions for data-informed decision-making [10, 21, 36]				✓	✓	✓						
Difficulty accessing or using online resources [9, 11-15, 17-19, 21, 22, 28, 31]	✓			✓			✓		✓			
Lack of infrastructure and technical support [8, 10, 12, 18, 20, 24, 25, 34, 37]	✓	✓	✓	✓	✓	✓	✓	✓	✓	✓	✓	✓
Inadequate resources [8-10, 14, 18, 36, 37]	✓		✓	✓		✓					✓	✓

	EVIDENCE SERVICE			DATA SERVICE			CAPACITY BUILDING SERVICE			PROJECT SUPPORT SERVICE		
	Identify, capture and process synthesised evidence	Translate into user friendly formats	Disseminate to decision-makers	Identify high risks and variations in practice	Translate into user friendly formats	Disseminate to decision-makers	Provide training in accessing and using evidence and data	Provide training in implementation & evaluation	Mentor and support	Provide advice regarding methodologies and methods	Assist with project development & administration	Assist with data capture, data entry and analysis
Negative attitudes or resistance to change [7, 8, 12, 20]		✓			✓		✓	✓				
Professional groups with different perspectives of evidence, knowledge base and skill set [32]								✓	✓			
Lack of triggers to initiate disinvestment discussions [30, 38-40]			✓			✓						
Lack of standardised processes for project delivery, responsibilities and accountability [23, 41, 42]								✓	✓	✓	✓	✓
Unrealistic project timelines [42]								✓	✓	✓	✓	✓
**ENABLERS**												
Training in use of evidence and data [11, 12, 17, 24, 34, 36]							✓	✓	✓	✓	✓	✓
Dissemination of research and data [9, 17, 36, 43, 44]			✓			✓						
Clarity, relevance, credibility and reliability of research findings [9, 17, 19, 45, 46]	✓	✓					✓					
Quality and timely data from health information systems [19, 21, 24]				✓	✓		✓					
Organisational willingness to invest in a knowledge translation culture [8, 36, 47]	✓	✓	✓	✓	✓	✓	✓	✓	✓	✓	✓	✓
Infrastructure or policy for accountability in knowledge use [8, 36, 37, 48, 49]			✓			✓						
Links to researchers or knowledge brokers [8, 9, 19, 47, 50]			✓			✓	✓	✓	✓	✓	✓	✓
Initiatives to integrate data into routine decision-making processes [43]				✓	✓	✓						

	EVIDENCE SERVICE			DATA SERVICE			CAPACITY BUILDING SERVICE			PROJECT SUPPORT SERVICE		
	Identify, capture and process synthesised evidence	Translate into user friendly formats	Disseminate to decision-makers	Identify high risks and variations in practice	Translate into user friendly formats	Disseminate to decision-makers	Provide training in accessing and using evidence and data	Provide training in implementation & evaluation	Mentor and support	Provide advice regarding methodologies and methods	Assist with project development & administration	Assist with data capture, data entry and analysis
**ADDITIONAL NEEDS**												
Capacity-building and provision of expertise and practical assistance [17, 21, 25, 35, 51-53]	✓	✓	✓	✓	✓	✓	✓	✓	✓	✓	✓	✓
New processes to use research and data 'proactively' to drive decisions [21, 34, 35, 52]	✓	✓	✓	✓	✓	✓						
Analysis, synthesis, interpretation and review of data in decision-making [21, 24, 34]				✓	✓	✓	✓	✓	✓	✓		✓
Incentives to change [30, 36, 41]										✓	✓	✓
Support to be tailored to units and professional needs [21, 46, 47]		✓			✓		✓	✓	✓	✓	✓	✓
Provision of a range of expertise in evaluation methods [34, 54]										✓	✓	✓
Support from others who had done the same or similar work to address feelings of isolation							✓	✓	✓	✓	✓	✓
**EVIDENCE-BASED INTERVENTIONS**												
Dissemination of summaries of systematic review evidence [28, 44, 55, 56]		✓	✓									
Tailored targeted messages [28, 44, 57-59]		✓	✓		✓	✓						
Training in critical appraisal [56, 58, 60]							✓		✓			
Interactive workshops [28, 60]							✓	✓	✓			
Multifaceted educational intervention [28, 60]							✓	✓	✓	✓	✓	✓

	EVIDENCE SERVICE [61]			DATA SERVICE			CAPACITY BUILDING SERVICE			PROJECT SUPPORT SERVICE		
	Identify, capture and process synthesised evidence	Translate into user friendly formats	Disseminate to decision-makers	Identify high risks and variations in practice	Translate into user friendly formats	Disseminate to decision-makers	Provide training in accessing and using evidence and data	Provide training in implementation & evaluation	Mentor and support	Provide advice regarding methodologies and methods	Assist with project development & administration	Assist with data capture, data entry and analysis
**BARRIERS**												
Lack of time and opportunity [7-20]			✓			✓					✓	✓
Lack of skills [7, 9, 11-15, 17, 19-26]	✓	✓	✓	✓	✓	✓	✓	✓	✓	✓	✓	✓
Lack of confidence [12, 27]	✓	✓	✓	✓	✓	✓	✓	✓	✓	✓	✓	✓
Lack of interest or competing priorities [14, 20, 27-29]			✓			✓	✓		✓			
Lack of awareness of research and data [7, 12, 17, 21, 25, 28]	✓		✓	✓		✓	✓					
Lack of use of available research and data [21, 24, 25, 28, 30]		✓	✓		✓	✓	✓	✓	✓	✓	✓	
Lack of relevant research and data [9, 10, 14-16, 18, 19, 21, 22, 31] particularly for disinvestment [19, 20, 23, 32, 33]	✓			✓			✓					
Poor quality of health data [10, 19, 21, 24, 31, 34, 35]				✓	✓	✓						
Unfamiliar or difficult to use formats of research and data [12, 17, 19-21, 24, 31]		✓			✓		✓					
Lack of policies and interventions for data-informed decision-making [10, 21, 36]				✓	✓	✓						
Difficulty accessing or using online resources [9, 11-15, 17-19, 21, 22, 28, 31]	✓			✓			✓		✓			
Lack of infrastructure and technical support [8, 10, 12, 18, 20, 24, 25, 34, 37]	✓	✓	✓	✓	✓	✓	✓	✓	✓	✓	✓	✓
Inadequate resources [8-10, 14, 18, 36, 37]	✓		✓	✓		✓					✓	✓
Negative attitudes or resistance to change [7, 8, 12, 20]		✓			✓		✓	✓				

	EVIDENCE SERVICE [61]			DATA SERVICE			CAPACITY BUILDING SERVICE			PROJECT SUPPORT SERVICE		
	Identify, capture and process synthesised evidence	Translate into user friendly formats	Disseminate to decision-makers	Identify high risks and variations in practice	Translate into user friendly formats	Disseminate to decision-makers	Provide training in accessing and using evidence and data	Provide training in implementation & evaluation	Mentor and support	Provide advice regarding methodologies and methods	Assist with project development & administration	Assist with data capture, data entry and analysis
Professional groups with different perspectives of evidence, knowledge base and skill set [32]								✓	✓			
Lack of triggers to initiate disinvestment discussions [30, 38-40]			✓			✓						
Lack of standardised processes for project delivery, responsibilities and accountability [23, 41, 42]								✓	✓	✓	✓	✓
Unrealistic project timelines [42]								✓	✓	✓	✓	✓
**ENABLERS**												
Training in use of evidence and data [11, 12, 17, 24, 34, 36]							✓	✓	✓	✓	✓	✓
Dissemination of research and data [9, 17, 36, 43]			✓			✓						
Clarity, relevance, credibility and reliability of research findings [9, 17, 19, 45, 46]	✓	✓					✓					
Quality and timely data from health information systems [19, 21, 24]				✓	✓		✓					
Organisational willingness to invest in a knowledge translation culture [8, 36, 47]	✓	✓	✓	✓	✓	✓	✓	✓	✓	✓	✓	✓
Infrastructure or policy for accountability in knowledge use [8, 36]			✓			✓						
Links to researchers or knowledge brokers [8, 9, 19, 47, 50]			✓			✓	✓	✓	✓	✓	✓	✓
Initiatives to integrate data into routine decision-making processes [43]				✓	✓	✓						
**ADDITIONAL NEEDS**												
Capacity-building and provision of expertise and practical assistance [17, 21, 25, 35, 51-53]	✓	✓	✓	✓	✓	✓	✓	✓	✓	✓	✓	✓

	EVIDENCE SERVICE [61]			DATA SERVICE			CAPACITY BUILDING SERVICE			PROJECT SUPPORT SERVICE		
	Identify, capture and process synthesised evidence	Translate into user friendly formats	Disseminate to decision-makers	Identify high risks and variations in practice	Translate into user friendly formats	Disseminate to decision-makers	Provide training in accessing and using evidence and data	Provide training in implementation & evaluation	Mentor and support	Provide advice regarding methodologies and methods	Assist with project development & administration	Assist with data capture, data entry and analysis
New processes to use research and data 'proactively' to drive decisions [21, 34, 35, 52]	✓	✓	✓	✓	✓	✓						
Analysis, synthesis, interpretation and review of data in decision-making [21, 24, 34]				✓	✓	✓	✓	✓	✓	✓		✓
Incentives to change [30, 36, 41]										✓	✓	✓
Support to be tailored to units and professional needs [21, 46, 47]		✓			✓		✓	✓	✓	✓	✓	✓
Provision of a range of expertise in evaluation methods [34, 54]										✓	✓	✓
Support from others who had done the same or similar work to address feelings of isolation							✓	✓	✓	✓	✓	✓
**EVIDENCE-BASED INTERVENTIONS**												
Dissemination of summaries of systematic review evidence [28, 55, 56]		✓	✓									
Tailored targeted messages [28, 57-59]		✓	✓		✓	✓						
Training in critical appraisal [56, 58, 60]							✓		✓			
Interactive workshops [28, 60]							✓	✓	✓			
Multifaceted educational intervention [28, 60]							✓	✓	✓	✓	✓	✓

FACTORS THAT INFLUENCED PROCESSES AND OUTCOMES OF SUPPORT SERVICES

CHARACTERISTICS OF THE DETERMINANTS OF EFFECTIVENESS*			ES[#]	DS	CBS	PSS
**External environment**	Political	Disinvestment was a priority topic for Department of Treasury which encouraged Department of Human Services to investigate it further	✓	✓	✓	✓
	Financial	Department of Human Services funding for SHARE enabled all the activities	✓	✓	✓	✓
		Withdrawal of funding in final year of program prevented implementation of some interventions and many of the evaluation activities		✗	✗	✗
**Organisation**	Financial	Monash Health funding for SHARE also enabled all the activities	✓	✓	✓	✓
		Monash Health funding for ES continued after Department of Human Services funding withdrawn	✓			
	Leadership	Support and endorsement was provided at senior levels (Board, Executive Management Team, Clinical Program Directors)	✓			
	Processes	Monash Health had multiple databases, housed with different custodians, with a range of methods of access; there was no coordination		✗		
		Evidence Service was implemented in a governance framework requiring mandatory responses from decision-makers	✓			
	Culture	Organisational (ES) and departmental (CBS) culture was supportive of evidence-based practice	✓		✓	
**Potential adopters**	Attitudes	Most target users viewed the proposals positively	✓		✓	✓
		Target users acknowledged their limitations, were enthusiastic about training and support and were willing to take advice and direction			✓	✓
		Committees declined support in accessing and using data		✗		
	Support	Pharmacy staff had support from management to attend training			✓	
	Leadership	Pharmacy staff, pharmacy-related committee members and SHARE pilot project teams demonstrated leadership by their participation			✓	✓
**Innovation**	Evidence	Developed from research and local data identifying barriers, enablers and expressed needs for content and format	✓	✓	✓	✓
		Good supporting evidence of effectiveness of chosen interventions	✓	✓	✓	✓
	Engagement and champions	Centre for Clinical Effectiveness has ownership of the project and authority to implement change	✓		✓	✓
		Centre for Clinical Effectiveness does not have ownership of the project and authority to implement change		✗		
	Compatibility with status quo	Within Centre for Clinical Effectiveness skill sets and priorities	✓		✓	✓
		Not within Centre for Clinical Effectiveness skill sets and priorities		✗		
		Proposal is not deliverable in original format (multiple often inaccessible datasets, lack of local capacity and capability)		✗		
	Trialability	All services were implemented in pilot mode and participants were informed that their feedback would be used to refine the processes	✓		✓	✓
		Implementing with small groups resulted in lack of critical mass for ongoing support services			✗	
**Implementation strategy**	Tailored to barriers and enablers	Barrier and enabler analysis focused on development of the innovation and not on development of implementation strategies	✗	✗	✗	
		Tailored to needs of individual projects and project teams				✓
	Knowledge and skills	Centre for Clinical Effectiveness team had skills in implementation of change	✓		✓	✓
		Health economist and health program evaluator engaged as consultants to the project team	✓		✓	✓
	Resources	Adequate resources initially	✓	✓	✓	✓
		Inadequate resources after Department of Human Services funding withdrawn		✗	✗	✗

* Not all factors from the taxonomy are listed, only those that influenced the pilot projects.
Some factors only influenced some of the support services.

[#] ES = Evidence Service, DS = Data Service, CBS = Capacity Building Service, PPS = Project Support Service
✓ = positive influence, ✗ = negative influence

REFERENCES

1. SurveyMonkey, . SurveyMonkey Inc., Palo Alto, California, USA. www.surveymonkey.com. Accessed March 2016.
2. Taylor R, Reeves B, Mears R, Keast J, Binns S, Ewings P et al. Development and validation of a questionnaire to evaluate the effectiveness of evidence-based practice teaching. Medical education. 2001;35(6):544-7.
3. NVivo qualitative data analysis software Version 8. QSR International Pty Ltd; 2008.
4. Michie S, Johnston M, Abraham C, Lawton R, Parker D, Walker A. Making psychological theory useful for implementing evidence based practice: a consensus approach. Quality & safety in health care. 2005;14(1):26-33. doi:10.1136/qshc.2004.011155.
5. Harris C, Allen K, Waller C, Brooke V. Sustainability in Health care by Allocating Resources Effectively (SHARE) 3: Examining how resource allocation decisions are made, implemented and evaluated in a local healthcare setting BMC health services research. 2017;(Details TBA).
6. Harris C, Green S, Ramsey W, Allen K, King R. Sustainability in Health care by Allocating Resources Effectively (SHARE) 1: Introducing a series of papers reporting an investigation of disinvestment in a local healthcare setting BMC health services research. 2017;(Details TBA).
7. Tricco AC, Cardoso R, Thomas SM, Motiwala S, Sullivan S, Kealey MR et al. Barriers and facilitators to uptake of systematic reviews by policy makers and health care managers: a scoping review. Implementation science : IS. 2016;11(1):4. doi:10.1186/s13012-016-0370-1.
8. Ellen ME, Leon G, Bouchard G, Ouimet M, Grimshaw JM, Lavis JN. Barriers, facilitators and views about next steps to implementing supports for evidence-informed decision-making in health systems: a qualitative study. Implementation science : IS. 2014;9(1):179. doi:10.1186/s13012-014-0179-8.
9. Oliver K, Innvar S, Lorenc T, Woodman J, Thomas J. A systematic review of barriers to and facilitators of the use of evidence by policymakers. BMC health services research. 2014;14(1):2. doi:10.1186/1472-6963-14-2.
10. van Panhuis WG, Paul P, Emerson C, Grefenstette J, Wilder R, Herbst AJ et al. A systematic review of barriers to data sharing in public health. BMC public health. 2014;14:1144. doi:10.1186/1471-2458-14-1144.
11. Clarke MA, Belden JL, Koopman RJ, Steege LM, Moore JL, Canfield SM et al. Information needs and information-seeking behaviour analysis of primary care physicians and nurses: a literature review. Health information and libraries journal. 2013;30(3):178-90. doi:10.1111/hir.12036.
12. Solomons NM, Spross JA. Evidence-based practice barriers and facilitators from a continuous quality improvement perspective: an integrative review. Journal of nursing management. 2011;19(1):109-20. doi:10.1111/j.1365-2834.2010.01144.x.
13. Younger P. Internet-based information-seeking behaviour amongst doctors and nurses: a short review of the literature. Health information and libraries journal. 2010;27(1):2-10. doi:10.1111/j.1471-1842.2010.00883.x.
14. Davies K, Harrison J. The information-seeking behaviour of doctors: a review of the evidence. Health information and libraries journal. 2007;24(2):78-94. doi:10.1111/j.1471-1842.2007.00713.x.
15. Nail-Chiwetalu B, Bernstein Ratner N. An assessment of the information-seeking abilities and needs of practicing speech-language pathologists. Journal of the Medical Library Association : JMLA. 2007;95(2):182-8, e56-7. doi:10.3163/1536-5050.95.2.182.
16. Dawes M, Sampson U. Knowledge management in clinical practice: a systematic review of information seeking behavior in physicians. International journal of medical informatics. 2003;71(1):9-15.
17. Dobbins M, DeCorby K, Twiddy T. A knowledge transfer strategy for public health decision makers. Worldviews on evidence-based nursing / Sigma Theta Tau International, Honor Society of Nursing. 2004;1(2):120-8. doi:10.1111/j.1741-6787.2004.t01-1-04009.x.
18. Bowen S, Erickson T, Martens PJ, Crockett S. More than "using research": the real challenges in promoting evidence-informed decision-making. Healthcare Policy. 2009;4(3):87-102.
19. Revere D, Turner AM, Madhavan A, Rambo N, Bugni PF, Kimball A et al. Understanding the information needs of public health practitioners: a literature review to inform design of an interactive digital knowledge management system. Journal of biomedical informatics. 2007;40(4):410-21. doi:10.1016/j.jbi.2006.12.008.
20. Niedzwiedzka BM. Barriers to evidence-based decision making among Polish healthcare managers. Health services management research : an official journal of the Association of University Programs in Health Administration / HSMC, AUPHA. 2003;16(2):106-15. doi:10.1258/095148403321591429.
21. Nutley T, Reynolds HW. Improving the use of health data for health system strengthening. Global health action. 2013;6:20001. doi:10.3402/gha.v6i0.20001.
22. Gilman IP. Evidence-based information-seeking behaviors of occupational therapists: a survey of recent graduates. Journal of the Medical Library Association : JMLA. 2011;99(4):307-10.
23. Marks L, Weatherly H, Mason A. Prioritizing investment in public health and health equity: what can commissioners do? Public health. 2013;127(5):410-8. doi:10.1016/j.puhe.2013.01.027.
24. Braa J, Heywood A, Sahay S. Improving quality and use of data through data-use workshops: Zanzibar, United Republic of Tanzania. Bulletin of the World Health Organization. 2012;90(5):379-84. doi:10.2471/blt.11.099580.
25. Robinson S, Williams I, Dickinson H, Freeman T, Rumbold B. Priority-setting and rationing in healthcare: evidence from the English experience. Social science & medicine. 2012;75(12):2386-93. doi:10.1016/j.socscimed.2012.09.014.
26. Cornelissen E, Mitton C, Davidson A, Reid RC, Hole R, Visockas AM et al. Changing priority setting practice: The role of implementation in practice change. Health policy (Amsterdam, Netherlands). 2014. doi:10.1016/j.healthpol.2014.04.010.

27. Finch E, Cornwell P, Ward EC, McPhail SM. Factors influencing research engagement: research interest, confidence and experience in an Australian speech-language pathology workforce. BMC health services research. 2013;13:144.
28. Wallace J, Byrne C, Clarke M. Improving the uptake of systematic reviews: a systematic review of intervention effectiveness and relevance. BMJ open. 2014;4(10):e005834. doi:10.1136/bmjopen-2014-005834.
29. Prendiville TW, Saunders J, Fitzsimons J. The information-seeking behaviour of paediatricians accessing web-based resources. Archives of disease in childhood. 2009;94(8):633-5. doi:10.1136/adc.2008.149278.
30. Polisena J, Clifford T, Elshaug AG, Mitton C, Russell E, Skidmore B. Case studies that illustrate disinvestment and resource allocation decision-making processes in health care: A systematic review. International journal of technology assessment in health care. 2013;29(2):174-84. doi:10.1017/s0266462313000068.
31. Vest JR, Kirk HM, Issel LM. Quality and integration of public health information systems: A systematic review focused on immunization and vital records systems. Online journal of public health informatics. 2012;4(2). doi:10.5210/ojphi.v4i2.4198.
32. Robert G, Harlock J, Williams I. Disentangling rhetoric and reality: an international Delphi study of factors and processes that facilitate the successful implementation of decisions to decommission healthcare services. Implementation science : IS. 2014;9(1):123. doi:10.1186/s13012-014-0123-y.
33. Rubinstein A, Belizan M, Discacciati V. Are economic evaluations and health technology assessments increasingly demanded in times of rationing health services? The case of the Argentine financial crisis. International journal of technology assessment in health care. 2007;23(2):169-76. doi:10.1017/s0266462307070274.
34. Jorm L. Routinely collected data as a strategic resource for research: priorities for methods and workforce. Public health research & practice. 2015;25(4):e2541540. doi:10.17061/phrp2541540.
35. Evans BA, Snooks H, Howson H, Davies M. How hard can it be to include research evidence and evaluation in local health policy implementation? Results from a mixed methods study. Implementation science : IS. 2013;8:17. doi:10.1186/1748-5908-8-17.
36. Stansfield SK, Walsh J, Prata N, Evans T. Information to Improve Decision Making for Health. In: Jamison DT, Breman JG, Measham AR, Alleyne G, Claeson M, Evans DB et al., editors. Disease Control Priorities in Developing Countries. Washington DC: The International Bank for Reconstruction and Development/The World Bank Group; 2006.
37. Gifford W, Davies B, Edwards N, Griffin P, Lybanon V. Managerial leadership for nurses' use of research evidence: an integrative review of the literature. Worldviews on evidence-based nursing / Sigma Theta Tau International, Honor Society of Nursing. 2007;4(3):126-45. doi:10.1111/j.1741-6787.2007.00095.x.
38. HealthPACT. Disinvestment in Australia and New Zealand. Health Policy Advisory Committee on Technology 2013. Available from: http://www.health.qld.gov.au/healthpact/docs/papers/workshop/disinvestment-report.pdf. Accessed: October 2016
39. Watt AM, Hiller JE, Braunack-Mayer AJ, Moss JR, Buchan H, Wale J et al. The ASTUTE Health study protocol: deliberative stakeholder engagements to inform implementation approaches to healthcare disinvestment. Implementation science : IS. 2012;7:101. doi:10.1186/1748-5908-7-101.
40. Elshaug AG, Hiller JE, Tunis SR, Moss JR. Challenges in Australian policy processes for disinvestment from existing, ineffective health care practices. Australia and New Zealand health policy. 2007;4:23. doi:10.1186/1743-8462-4-23.
41. Mitton C, Dionne F, Donaldson C. Managing healthcare budgets in times of austerity: the role of program budgeting and marginal analysis. Applied health economics and health policy. 2014;12(2):95-102. doi:10.1007/s40258-013-0074-5.
42. Hughes E, McKenny K. Decommissioning and Disinvestment Toolkit 2013-2014 Castle Point and Rochford Clinical Commissioning Group 2013. Available from: http://castlepointandrochfordccg.nhs.uk/about-us/key-documents/policies/corporate-policies/299-decommissioning-and-disinvestment-strategy/file. Accessed: October 2016
43. Morrato EH, Elias M, Gericke CA. Using population-based routine data for evidence-based health policy decisions: lessons from three examples of setting and evaluating national health policy in Australia, the UK and the USA. Journal of public health (Oxford, England). 2007;29(4):463-71. doi:10.1093/pubmed/fdm065.
44. Chambers D, Wilson PM, Thompson CA, Hanbury A, Farley K, Light K. Maximizing the impact of systematic reviews in health care decision making: a systematic scoping review of knowledge-translation resources. The Milbank quarterly. 2011;89(1):131-56. doi:10.1111/j.1468-0009.2011.00622.x.
45. Kosteniuk JG, Morgan DG, D'Arcy CK. Use and perceptions of information among family physicians: sources considered accessible, relevant, and reliable. Journal of the Medical Library Association : JMLA. 2013;101(1):32-7. doi:10.3163/1536-5050.101.1.006.
46. Cilenti D, Brownson RC, Umble K, Erwin PC, Summers R. Information-seeking behaviors and other factors contributing to successful implementation of evidence-based practices in local health departments. Journal of public health management and practice : JPHMP. 2012;18(6):571-6. doi:10.1097/PHH.0b013e31825ce8e2.
47. Riley BL, Robinson KL, Gamble J, Finegood DT, Sheppard D, Penney TL et al. Knowledge to action for solving complex problems: insights from a review of nine international cases. Health promotion and chronic disease prevention in Canada. 2015;35(3):47-53.
48. Fronsdal KB, Facey K, Klemp M, Norderhaug IN, Morland B, Rottingen JA. Health technology assessment to optimize health technology utilization: using implementation initiatives and monitoring processes. International journal of technology assessment in health care. 2010;26(3):309-16. doi:10.1017/s0266462310000309.
49. Healy J, Braithwaite J. Designing safer health care through responsive regulation. The Medical journal of Australia. 2006;184(10 Suppl):S56-9.
50. Giles-Corti B, Sallis JF, Sugiyama T, Frank LD, Lowe M, Owen N. Translating active living research into policy and practice: One important pathway to chronic disease prevention. Journal of public health policy. 2015;36(2):231-43. doi:10.1057/jphp.2014.53.

51. Henshall C, Schuller T, Mardhani-Bayne L. Using health technology assessment to support optimal use of technologies in current practice: the challenge of "disinvestment". International journal of technology assessment in health care. 2012;28(3):203-10. doi:10.1017/s0266462312000372.
52. Schmidt DE. The development of a disinvestment framework to guide resource allocation decisions in health service delivery organizations. The University of British Columbia 2010. Available from: https://open.library.ubc.ca/cIRcle/collections/ubctheses/24/items/1.0073252. Accessed: October 2016
53. Williams I, Bryan S, McIver S. The use of economic evaluations in NHS decision making: A review and empirical investigation: Health Economics Facility, University of Birmingham, Health Services Management Centre, University of Birmingham, 2006.
54. Bowers B, Cohen LW, Elliot AE, Grabowski DC, Fishman NW, Sharkey SS et al. Creating and supporting a mixed methods health services research team. Health services research. 2013;48(6 Pt 2):2157-80. doi:10.1111/1475-6773.12118.
55. Murthy L, Shepperd S, Clarke MJ, Garner SE, Lavis JN, Perrier L et al. Interventions to improve the use of systematic reviews in decision-making by health system managers, policy makers and clinicians. Cochrane database of systematic reviews (Online). 2012;9:CD009401. doi:10.1002/14651858.CD009401.pub2.
56. Wallace J, Byrne C, Clarke M. Making evidence more wanted: a systematic review of facilitators to enhance the uptake of evidence from systematic reviews and meta-analyses. International journal of evidence-based healthcare. 2012;10(4):338-46. doi:10.1111/j.1744-1609.2012.00288.x.
57. LaRocca R, Yost J, Dobbins M, Ciliska D, Butt M. The effectiveness of knowledge translation strategies used in public health: a systematic review. BMC public health. 2012;12:751. doi:10.1186/1471-2458-12-751.
58. Moore G, Redman S, Haines M, Todd A. What works to increase the use of research in population health policy and programmes: a review. Evidence and Policy: A Journal of Research, Debate and Practice. 2011;7(3):277-305. doi:10.1332/174426411X579199.
59. Perrier L, Mrklas K, Lavis JN, Straus SE. Interventions encouraging the use of systematic reviews by health policymakers and managers: a systematic review. Implementation science : IS. 2011;6:43. doi:10.1186/1748-5908-6-43.
60. Young T, Rohwer A, Volmink J, Clarke M. What Are the Effects of Teaching Evidence-Based Health Care (EBHC)? Overview of Systematic Reviews. PLoS One. 2014;9(1):e86706. doi:10.1371/journal.pone.0086706.
61. Harris C, Garrubba M, Melder A, Voutier C, Waller C, King R et al. Sustainability in Health care by Allocating Resources Effectively (SHARE) 8: Developing, implementing and evaluating an Evidence Dissemination Service in a local healthcare setting. BMC health services research. 2017;(Details TBA).
